# *De novo* Whole-Genome Assembly of *Moringa oleifera* Helps Identify Genes Regulating Drought Stress Tolerance

**DOI:** 10.3389/fpls.2021.766999

**Published:** 2021-12-14

**Authors:** P Sushree Shyamli, Seema Pradhan, Mitrabinda Panda, Ajay Parida

**Affiliations:** ^1^Institute of Life Sciences, An Autonomous Institute Under Department of Biotechnology Government of India, NALCO Square, Bhubaneswar, India; ^2^Regional Centre for Biotechnology, NCR Biotech Science Cluster, Faridabad, India

**Keywords:** *Moringa oleifera*, whole-genome sequencing (WGS), abiotic stress, HSFs, differential expression

## Abstract

Abiotic stresses, especially drought stress, are responsible for heavy losses in productivity, which in turn poses an imminent threat for future food security. Understanding plants’ response to abiotic stress at the molecular level is crucially important for mitigating the impacts of climate change. *Moringa oleifera* is an important multipurpose plant with medicinal and nutritional properties and with an ability to grow in low water conditions, which makes the species an ideal candidate to study the regulatory mechanisms that modulate drought tolerance and its possible use in agroforestry system. In the present communication, we report whole-genome sequencing (WGS) of this species and assemble about 90% of the genome of *M. oleifera* var. Bhagya into 915 contigs with a N50 value of 4.7 Mb and predicted 32,062 putative protein-coding genes. After annotating the genome, we have chosen to study the heat shock transcription factor (HSF) family of genes to analyze their role in drought tolerance in *M. oleifera*. We predicted a total of 21 HSFs in the *M. oleifera* genome and carried out phylogenetic analyses, motif identification, analysis of gene duplication events, and differential expression of the HSF-coding genes in *M. oleifera*. Our analysis reveals that members of the HSF family have an important role in the plant’s response to abiotic stress and are viable candidates for further characterization.

## Introduction

*Moringa oleifera*, which belongs to the family Moringaceae, is native to the Indian subcontinent and naturalized in tropical and subtropical regions around the world. The genus *Moringa* has 13 species (Gandji et al., 2018), of which two species, *viz.*, *M. oleifera* Lam and *M. concanensis* Nimmo, are found in India (Pandey et al., 2011). *M. oliefera* is a versatile plant and a repository of essential phytochemicals such as tannins, sterols, terpenoids, flavonoids, saponins, anthraquinones, alkaloids, and reducing sugar, present in leaves, pods, and seeds (Gopalakrishnan et al., 2016). Apart from its nutritional properties, *M. oleifera* is also known for its ability to grow in semi-arid environments. The two most common abiotic stresses, namely, hypersalinity and drought, are responsible for the majority of crop loss worldwide. Plants engage in various cellular mechanisms to deal with such extreme conditions. One of the most common mechanisms is the production of osmolytes such as proline (Liang et al., 2013). The ultimate consequence is the enhanced production of reactive oxygen species (ROS) under prolonged or severe water deficit (Hayat et al., 2012). ROS production causes damage through membrane peroxidation. Polyphenols, which are antioxidants and produced by plants, help to retain ROS at less damaging levels. Part of the *M. oleifera* has medicinal attributes because of the possession of these antioxidants (Sofidiya et al., 2006).

The accumulation of secondary metabolites is believed to be strongly dependent on the growing conditions (Lommen et al., 2008). Polyphenols and proline often demonstrate various bioactivities such as guarding the cell against stress, involvement in metal chelation (Sharma and Dietz, 2009), and functioning as a ROS scavenger (Liang et al., 2013). An extensive range of experiments have elucidated that plants exposed to drought stress accumulate higher concentrations of secondary metabolites, antioxidants, and proline. Increased concentrations of phenols as well as nitrogen-containing substances are reported in almost all classes of natural products such as alkaloids, cyanogenic glucosides, or glucosinolates (Selmar and Kleinwächter, 2013). High concentration of cellular proline of up to 80% of amino acid pool has been reported under stress and as low as 5% under normal conditions (Delauney and Verma, 1993). It is evident that water stress plays a role in the accumulation pattern and concentrations of these compounds. Secondary metabolites have become relevant due to their crucial applications in medicinal, nutritive, and cosmetic purposes as well as their importance in plant stress physiology (Edreva et al., 2008).

A recent study investigated the presence of polyphenolic compounds, antioxidant activities, proline accumulation, and their distribution in the different parts of *M. oleifera* plant under varying water regimes. Results from the research indicated that drought stress influences the synthesis and concentration of the osmolyte proline and polyphenolic compounds in *M. oleifera* plant. Their findings showed that under water scarcity, *M. oleifera* responds by an upsurge biosynthesis and accumulation of phenolic, condensed tannin, and proline contents in all plant parts but mostly in leaves (Chitiyo et al., 2021). This indicates that accumulating osmoprotectants and antioxidant compounds such as phenolic compounds are some of the mechanisms employed by *M. oleifera* to cope up with drought-induced oxidative stress and dehydration.

Therefore, *M. oleifera* represents a promising species capable of minimizing the adverse effects of drought stress and can enhance the soil of arid regions (Boumenjel et al., 2020). In this study, we have undertaken *de novo* assembly and annotation of *M. oleifera* var Bhagya. A comparison of its genome with genomes of other plants revealed a number of orthologous groups that are important for growth and survival of plants. We have also identified the members of heat shock transcription factors (HSFs) in the genome of *M. oleifera* and analyzed their expression in response to drought stress.

## Materials and Methods

### Plant Material, Growth Conditions, and Stress Treatment

Seeds for four varieties of *M. oleifera*, namely, Bhagya, ODC3, PKM1, and PKM2, were collected from Krishi Vigyan Kendra, Dhenkanal, Odisha. The seeds were soaked in distilled water overnight and planted in a mix of soil rite and vermicompost in a ratio of 3:1. Plants were grown under controlled condition: 14 h/10 h: light/dark; 28°C/25°C ± 2; 65% RH, for 4 weeks. Young leaves of *M. oleifera* var. Bhagya were collected for high-quality DNA isolation for whole-genome sequencing (WGS). We subjected the 30-day-old young plants of *M. oleifera* to drought stress by withholding water for different time periods. We observed noticeable physical changes in the plants after 10 days. However, prolonged stress affected the quality of tissue and the subsequent RNA isolation yielded poor quality. Therefore, we decided to use the tissues after 10 days of drought stress (Supplementary Figure 1). Plants in control conditions were watered regularly as per requirement. Leaves and root tissue samples were collected from control and stress-treated plants, frozen in liquid nitrogen, and stored at −80°C. Three biological replicates were collected for each sample group.

### High-Quality Genomic DNA Extraction and Whole-Genome Sequencing

High molecular weight genomic DNA was isolated from the young leaves of *M. oleifera* var. Bhagya using the CTAB-based method (Doyle, 1991) with some modifications. DNA libraries were prepared for sequencing on PacBio Sequel and Illumina HiSeq 2500 platforms to generate long and short reads, respectively (AgriGenome Labs Pvt. Ltd, India). Raw reads were filtered to remove low-quality reads with FastQCv 0.11.9 with default parameters.^1^

### Genome Assembly and Annotation

The cleaned reads were subjected to Kmergenie (Chikhi and Medvedev, 2014) to predict the optimal k-value and assembly size that were found to be 96 and 392,308,674 bp, respectively. *De novo* assembly was performed at AgriGenome Pvt. Ltd^2^ using MaSuRCA (Zimin et al., 2013), and the assembly with the default k-mer sizes using both long and short reads was selected to be optimal. The repeat sequences from the assembled genome were masked using RepeatMasker V2.2 (Tempel, 2012) with *Arabidopsis* model. CDSs were predicted from the masked assembly using Augustus V3.3.1 (Stanke et al., 2004) with *Arabidopsis* trained model. We found 32,062 predicted genes in the assembly.

The predicted genes were annotated using our in-house pipeline. The predicted genes were compared with Uniprot database using BLASTX program with E-value cutoff of 10^–5^. The best BLASTX hit based on query coverage, identity, similarity score, and description of each gene was filtered out. The top BLASTX hit of each gene and the organism name was extracted. The gene ontology (GO) terms Molecular Function (MF), Cellular Component (CC), and Biological Process (BP) for genes were mapped using the current annotation sets^3^ and GO Slim terms.^4^

### Repeat Elements, Non-coding Regions, and Quality Assessment for Genome Assembly

We identified repetitive elements through both RepeatModeler v.1.0.10,^5^ which employed RECON and RepeatScout to predict interspersed repeats, and then obtained the consensus repeat library. The Tandem Repeat Finder software^6^ was used to identify the tandem repeats with the parameters: 2 7 7 80 10 50 2000 -l 3 -m. LTR-RTs were identified with LTR_FINDER_parallel (Ou and Jiang, 2019) with default parameters.

Non-coding RNAs were predicted as follows: tRNAscan-SE v 2.0 software (Lowe and Eddy, 1997) was used to predict the tRNAs with eukaryotic parameters. miRNAs and snRNAs were detected using Infernal cmscan v 1.1.4 (Nawrocki and Eddy, 2013) to search the Rfam database (Griffiths-Jones et al., 2005). The ncRNA database of *Arabidopsis thaliana* was downloaded from Phytozome v 12.1^7^ and used for annotating the rRNA in *M. oleifera* genome sequence.

We used two strategies to assess the quality of genome assembly: BUSCO alignment and LTR Assembly index (LAI). The BUSCO pipeline^8^ used datasets from the plant lineage (EmbryophytaOrthoDB release 9; Kriventseva et al., 2015) to assess the number of complete BUSCOs represented in the *M. oleifera* genome. LAI uses LTR-RTs to evaluate assembly continuity and has been widely used to evaluate the assembly quality of plant genomes with high contents of repetitive sequences. The LTR-RTs identified in this study were used as input for LTR_retriever (Ou et al., 2018) to calculate LAI.

### Ortho Group Inference

Amino acid sequences for primary transcripts of 11 plant species, namely, *A. thaliana, Brassica oleracea, Carica papaya, Cicer arietinum, Citrus sinensis, Medicago truncatula, Oryza sativa, Populus trichocarpa, Ricinus communis, Theobroma cacao*, and *Vitis vinifera*, were downloaded from Phytozome v 12.1 (see text footnote 7). Primary transcripts for *M. oleifera* were determined with TransDecoder^9^ and produced 23,744 genes. Orthologous groups were determined using OrthoFinder v. 2.2.0 (Emms and Kelly, 2019). The data available in the results of this analysis were used to build the species tree using FigTree.^10^

### The RNA-Seq Assembly of *Moringa oleifera* Transcriptome and Differential Gene Expression

The RNA-Seq reads for various tissues of M. *oleifera* var. Bhagya were downloaded from NCBI SRA database (see “Data availability”) and filtered with Trimmomatic-0.39 with default parameters (ILLUMINACLIP:TruSeq3-PE-2.fa:2:30:10 LEADING:3 TRAILING:3 SLIDINGWINDOW:4:15 MINLEN:36-threads 8) (Bolger et al., 2014). The high-quality reads were assembled into transcripts using the “–genome_guided_bam” feature of Trinity software (Grabherr et al., 2011), which utilized the RNA-Seq reads aligned to the *M. oleifera* genome reported in this study as input. We removed redundant transcripts and retained the longest coding representative of each transcript using cdhit (cdhit-est-c 0.98; Li and Godzik, 2006) and TransDecoder v 5.5.0 (see text footnote 9). We also used control and drought-stressed tissue samples (leaves and root) of *M. oleifera* var. Bhagya for RNA-seq. Total RNA was extracted from frozen plant tissues using RNA extraction kit (STRN50, Sigma Aldrich, St. Louis, MO, United States) following the manufacturer’s protocol. RNA concentration and purity of each sample were confirmed using Nanodrop 2000 Spectrophotometer (Thermo Scientific, Wilmington, DE, United States). The integrity of extracted RNA was also ensured by resolving on 1.2% agarose gel containing 18% formaldehyde and checking on automated electrophoresis system (Agilent 4200 TapeStation, Agilent, United States). Samples with a RIN > 7 were taken for library preparation. The libraries were prepared taking three biological replicates of each sample (Control_Leaf, Control_Root, Drought_Leaf and Drought_Root) using the TruSeq Stranded mRNA Library Prep kit (Illumina, United States). After quality assessment, the libraries were pooled and sequenced on NextSeq550 platform (Illumina, United States). The reads obtained were demultiplexed with bcl2fastq software (Illumina, United States) and filtered for quality and adapters with Trimmomatic-0.39 (ILLUMINACLIP:TruSeq3-PE-2.fa:2:30:10 LEADING:3 TRAILING:3 SLIDINGWINDOW:4:15 MINLEN:36-threads 8) (Bolger et al., 2014). The filtered reads were mapped onto the assembled transcriptome of *M. oleifera* using Bowtie2 (Langmead and Salzberg, 2012), and the *in silico* expression profile was generated with RSEM (RNA-Seq by Expectation–Maximization^11^) and edgeR.^12^ Gene enrichment for the differentially expressed genes was carried out with KOBAS^13^ and AgriGO with *A. thaliana* as reference using the Singular Enrichment Analysis (SEA) tool.^14^

### Identification of Heat Shock Transcription Factors in *Moringa oleifera* Genome

In order to be inclusive, we followed two strategies to identify all HSFs in the *M. oleifera* genome. First, we downloaded amino acid sequences of HSFs for all species from PlantTFDB^15^ and used BlastP^16^ to search protein sequences of *M. oleifera* against them to identify HSFs based on homology (with an e-value of 10^–5^, choosing the best alignment). Then, we downloaded the HMM profile for DNA-binding domain (DBD) of HSFs from Pfam (PF00447) and used the HMM profile to identify DBD in those HSFs identified in the previous step. All sequences that did not contain a DBD were removed. Finally, we removed redundancy by aligning the protein sequences and removed duplicate sequences to get a final list of 21 HSFs in *M. oleifera.*

### Nomenclature and Classification of Heat Shock Transcription Factors

The HSFs were mapped onto the scaffolds in whole-genome assembly of *Moringa* and named serially as MolHSF1-MolHSF21 according to their mapped position on the genome contigs. The HSFs were classified into three classes according to their similarities to the AtHSFs. The coiled coil (heptad or HR-A/B) domains were predicted using Marcoil software.^17^ Although MolHSFs 9, 11, and 13 did not show the presence of coiled coil structure (which is one of the requirements for a protein to qualify as a HSF), they have been included in this study due to their unusually long HSF-DBD. ProtParam tool in ExPASy (expasy.org) was used to determine pI of the peptides, and NLS were predicted using NLStradamus^18^ and NLSMapper^19^ and nuclear export signals (NESs) with,^20^ respectively.

### Phylogenetic Analysis and Conserved Domain Identification

The amino acid sequences of the 21 MolHSFs were aligned by ClustalW and phylogenetic tree were constructed using MEGAX software (Kumar et al., 2018) with neighbor-joining method, Poisson correction, and 1,000 bootstraps. Domains were identified using Pfam and SMART databases and visualized using DOG 2.0.^21^ We used MEME^22^ to identify conserved motifs in the MolHSF protein sequences, and gene structure was analyzed using GSDS.^23^ The protein sequences of HSFs in *Moringa* were compared to those from *Arabidopsis*, *T. cacao, C. papaya*, and *O. sativa*. The evolutionary history was inferred by using the maximum-likelihood method and JTT matrix-based model. The multiple sequence alignments were visualized with JalView^24^ (Waterhouse et al., 2009).

### Analysis of Duplication Events and Promoter Sequence

The duplication events for MolHSFs were identified using default parameters of MCScanX software (Wang et al., 2012). The ratios of non-synonymous (Ka) and synonymous (Ks) substitutions were calculated with TBtools (Chen et al., 2020). Promoter sequences were isolated by extracting 2,000 bp sequence upstream of TSS of the MolHSFs using BEDTools (Quinlan and Hall, 2010). Conserved motifs were identified with PlantCARE.^25^

### Isolation of RNA and Quantitative Real-Time PCR

The relative expression of the selected HSFs was quantified using qRT-PCR. The FASTA sequences of the selected transcripts were retrieved, and primers were designed using Integrated DNA Technologies (IDT) Primer design online tool by selecting the generic option and the following criterion: amplicon size of 100–150 bp; primer length of 18–23 bases; melting temperature of 57–63°C; and GC content of 40%–60% (Supplementary Table 7).

For each RNA sample, 1 μg of total RNA was treated with RNAse-free DNase 1 (Sigma-AMPD1, St. Louis, United States) and reverse-transcribed to synthesize cDNA using the first-strand cDNA synthesis kit (K1612, Thermo Scientific, MA, United States). The qRT-PCR was performed on QuantStudio-5 real-time PCR system (Thermo Fisher Scientific, United States) with SYBR green chemistry (Applied Biosystems, United States) in three technical and two biological replicates. The expression was normalized by housekeeping gene actin since we observed the stable expression of *M. oleifera* actin in our analysis (Ct values were in the range of 21–22). Each reaction (5 μl SYBR Green, 1 μl template cDNA, 1 μl each of the primers (10μM), and 2 μl RNase-free water) was performed three times with the following program: 50°C (2 min), 95°C (10 min) followed by 40 cycles of 95°C (15 s), 53°C (1 min), and melt curve stage of 95°C (15 s) and 65°C (15 s). The expression values were calculated using the comparative 2^–ΔΔCt^ method. The qRT-PCR analysis is presented graphically by taking the RQ values. Correlation between the log_2_ fold change values of *in silico* and qRT-PCR gene expression data was charted on MS Office Excel.

## Results

### Assembly and Annotation of *Moringa oleifera* Genome

Sequencing the genome of *M. oleifera* var. Bhagya generated 23.02 Gb of total reads on the SMRT platform and 90.9 Gb of short reads on the Illumina sequencing platform (Supplementary Table 1). This produced a cumulative coverage of more than 300× since the reported genome size of *Moringa* is 315 Mb (Tian et al., 2015). The clean reads were assembled into 915 contigs with MaSuRCa (Zimin et al., 2013) and yielded a genome assembly with an N50 value of 4.7 Mb, the longest contig length of 13.8 Mb, and representing about 281 Mb (∼90% of total genome) of *Moringa* genome (Table 1).

**TABLE 1:** Attributes of the whole-genome assembly of *M. oleifera*.

***De novo* genome assembly**			
Total number of contigs			915
Length of the longest contig			13,807,473 bp
Total length			281,946,330 bp
N50			4,719,167 bp
Number of N’s per 100 kbp			0.25
GC%			37.82
**Annotation**	
Number of predicted protein-coding genes			31,056
Average gene length (bp)			1,842
Average CDS length (bp)			1,798

**Repeat elements**			

**Type**	**Number of elements**	**Length occupied (bp)**	**Percentage of sequence**

Retroelements	4,524	1,949,564	0.69
SINEs	1	100	0.00
Penelope	0	0	0.00
LINEs	357	54,439	0.02
CRE/SLACS	0	0	0.00
L2/CR1/Rex	0	0	0.00
R1/LOA/Jockey	0	0	0.00
R2/R4/NeSL	0	0	0.00
RTE/Bov-B	26	3,871	0.00
L1/CIN4	331	50,568	0.02
LTR elements	4,166	1,895,025	0.67
BEL/Pao	0	0	0.00
Ty1/Copia	2,521	1,269,453	0.45
Gypsy/DIRS1	1,636	624,847	0.22
Retroviral	0	0	0.00
DNA transposons	2,783	443,823	0.16
hobo-Activator	170	41,476	0.01
Tc1-IS630-Pogo	37	1,820	0.00
En-Spm	0	0	0.00
MuDR-IS905	0	0	0.00
PiggyBac	0	0	0.00
Tourist/Harbinger	81	18,723	0.01
Other (Mirage,P-element, Transib)	0	0	0.00
Rolling circles	329	31,500	0.01
Unclassified	171,757	142,408,786	50.51
Total interspersed repeats		144,802,173	51.36
Small RNA	473	58,633	0.02
Satellites	69	7,634	0.00
Simple repeats	63,799	2,641,831	0.94
Low complexity	15,520	779,798	0.28

The BUSCO analysis revealed that 95.9% of core genes from the Embryophyta were present in the *M. oleifera* genome, of which 94.4% were complete and single-copy BUSCOs (Supplementary Table 2). In addition to that, LTR assembly index (LAI) score of 10.27 is an indication of a good-quality genome assembly (Supplementary Table 2).

**FIGURE 1:**
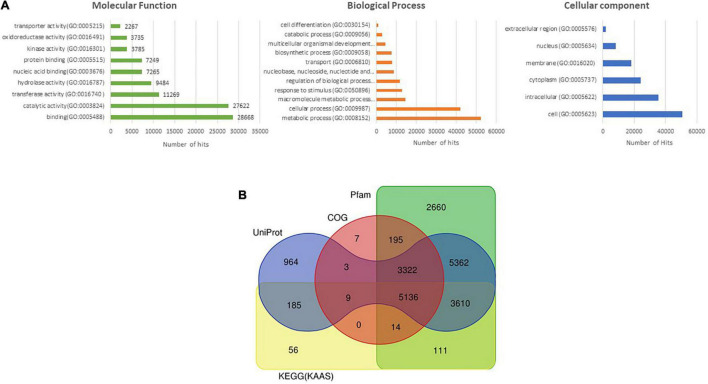
**(A)** Assignment of Gene ontology (GO) terms to predicted protein-coding genes of *M*. *oleifera*. **(B)** Venn diagram depicting the annotation of protein-coding sequences of *M. oleifera* with various databases.

A total of 32,062 protein-coding genes were predicted from the assembled genome, which came down to 31,056 after removing redundancy. Gene ontology terms were assigned using the annotations from UniProt/Swiss-Prot database and processing them through in-house pipelines. Processes related to “binding” and “catalytic activity” were over-represented in the molecular functions category, while biological processes such as “cellular” and “metabolic processes” were most abundantly represented (Figure 1A). These sequences were searched against the UniProt/Swiss-Prot database,^26^ Pfam database,^27^ KEGG,^28^ and COG databases,^29^ which annotated 21,634 of the predicted genes (Figure 1B).

There are two previous reports of WGS and assembly of *M. oleifera*. Tian et al. (2015) reported 33,332 contigs with N50 value of 1.14 Mb and a total of 19,465 protein-coding genes, while Chang et al. (2018) reduced the number of assembled contigs to 22,329 albeit with a N50 of 0.9 Mb and predicted 18,451 protein-coding genes. In comparison with the assembly reported in this study, we assembled the genome to 915 contigs with the N50 value of 4.7 Mb and 31,056 non-redundant protein-coding genes. We also used a more recently available whole-genome assembly for *M. oleifera* in the database of NCBI (PRJNA268707), along with the one reported by Chang et al. (2018) to perform a more exhaustive comparison between the assemblies (Supplementary Table 3). All data presented in this report point to the better quality and accuracy of the genome assembly reported for *M. oleifera*.

### Orthologous Groups and Important Gene Families

**FIGURE 2:**
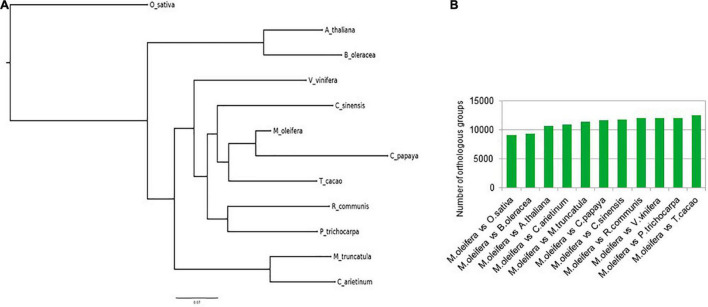
**(A)** Ortho group inference using FigTree. *M. oleifera* had more similarity with *C. papaya* and *T. cacao*, while forming a separate clade from *A. thaliana* and *B. oleracea*. **(B)** Number of orthologs shared by *M. oleifera* with the various plants.

The course of evolution has ensured the conservation of gene families important for survival of organisms. One of the methods to determine these gene families is to identify the orthologous groups after a phylogenetic comparison of the protein sequences in a group of varied organisms. We identified orthogroups after comparing a total of 378,983 protein sequences from 12 different plants, namely, *M. oleifera, A. thaliana, B. oleracea, C. papaya, C. arietinum, C. sinensis, M. truncatula, O. sativa, P. trichocarpa, R. communis, T. cacao*, and *V. vinifera.* Of the 6,574 orthogroups that contained representative protein sequences from all 12 species, a total of 536 were single-copy orthogroups (Supplementary Table 4). The species tree constructed with the data from this analysis revealed that *M. oleifera* had more similarity with *C. papaya* and *T. cacao*, while forming a separate clade from *A. thaliana* and *B. oleracea* (Figure 2A). These results are also reflected in the number of orthologs shared by *M. oleifera* with the various plants. It shares the most orthologs with *T. cacao*, while sharing the least with *O. sativa* (Figure 2B). The protein sequences for all plants in the single-copy orthologous groups were annotated against the protein sequences of *A. thaliana* in the UniProt/Swiss-Prot database using BlastP to identify the important gene families. The analysis revealed that a number of ion channel transporters, pentatricopeptide repeat-containing proteins, chaperones, and transcription factors such as WRKY, HSFs, and CCCH zinc finger transcription factors are conserved, indicating their importance in plant growth and survival (Supplementary Table 5).

### Simple Sequence Repeats in *Moringa oleifera* Genome

Simple sequence repeats (SSRs) are one of the most widespread molecular markers in plant genomes and have proven to be the popular choice for estimating genetic diversity and molecular breeding (Pan et al., 2020). A total of 92,163 SSRs were identified in 594 out of the 915 contigs of the assembled genome of *M. oleifera* (Table 2). Tetranucleotide repeats were most abundant followed by di- and trinucleotide repeats. Genic SSRs are especially helpful in assaying functional diversity and marker-assisted selection (MAS) in plants (Li et al., 2021). Therefore, we also mined SSRs in the protein-coding sequences of *M. oleifera* and identified 3,362 SSRs in 31,056 protein-coding genes, with the trinucleotide repeats being most abundant (Table 2). We have designed primers targeting genic SSRs, which can be used as a valuable resource for crop improvement (Supplementary Table 6).

**TABLE 2:** Results of microsatellite (SSRs) identification in *M. oleifera*.

**SSRs in whole genome**	
Total number of sequences examined	915
Total size of examined sequences (bp)	281,946,330
Total number of identified SSRs	92,163
Number of SSR-containing sequences	594
Number of sequences containing more than 1 SSR	465
Number of SSRs present in compound formation	10,902

**Distribution to different repeat-type classes**	

**Unit size**	**Number of SSRs**

Dinucleotide repeats	27,200
Trinucleotide repeats	17,276
Tetranucleotide repeats	31,789
Pentanucleotide repeats	10,734
Hexanucleotide repeats	5,164
**SSRs in protein-coding genes**	
Total number of sequences examined	31,056
Total size of examined sequences (bp)	57,197,102
Total number of identified SSRs	3,632
Number of SSR-containing sequences	3,109
Number of sequences containing more than 1 SSR	366
Number of SSRs present in compound formation	88

**Distribution to different repeat-type classes**	

**Unit size**	**Number of SSRs**

Dinucleotide repeats	131
Trinucleotide repeats	1,815
Tetranucleotide repeats	1,414
Pentanucleotide repeats	109
Hexanucleotide repeats	163

### Transcriptome of *Moringa oleifera* and Differential Expression of Genes in Response to Drought Stress

**FIGURE 3:**
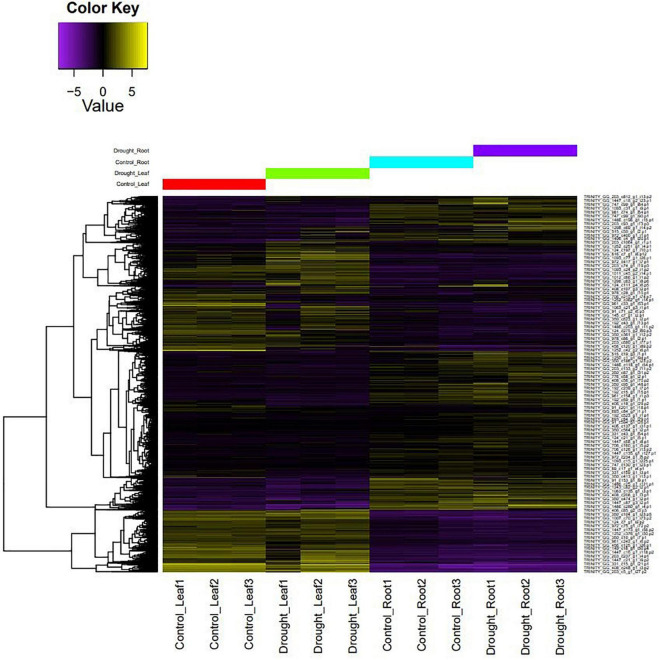
A representative heat map of differentially expressed genes under control *vs.* drought conditions.

The filtered, high-quality reads from leaf, root, stem, flower, and pod tissues of *M. oleifera* var. Bhagya were mapped onto the genome to generate a genome-guided transcriptome. We obtained 1,38,362 assembled transcripts, which we reduced to 1,37,132 transcripts after removing redundancy and retaining the longest CDS for each transcript. The assembly statistics and quality assessment suggest that this transcriptome assembly is of good quality (Supplementary Table 8). RNA-Seq of the control and drought-treated leaf and root tissues of *M. oleifera* was done. The RNA-Seq generated about 753.5 million reads which were filtered for quality. About 711 million reads (94.37%) were retained after removing low quality reads and adapter sequences. We mapped the high-quality reads from each sample onto the transcriptome of *M. oleifera* assembled in this study to develop a representative heat map of differentially expressed genes (Figure 3). Overall, there was a more pronounced effect of drought on leaves as compared to roots. A homology-based search against the UniProt/Swiss-Prot database revealed a number of differentially expressed genes to be transcription factors/regulators. These included Zn finger-containing transcription factors, ethylene-responsive transcription factors, WRKY, bHLH, and a number of transporters and genes associated with secondary metabolite production. In addition to these, we also found a number of heat shock proteins (HSPs) and HSFs to be differentially expressed in response to drought stress (Supplementary Table 9). Enrichment analysis revealed the over-representation of genes related to “Biosynthesis of secondary metabolites” and “response to stimuli.” This provided us with an idea of the regulatory mechanisms regulating drought stress response in *M. oleifera* (Supplementary Figure 2).

### Heat Shock Transcription Factors in the *Moringa oleifera* Genome

Heat shock transcription factors have emerged as important regulators of response to abiotic stress in plants, and although *Moringa* is mainly valued for its medicinal properties, the fact that it can withstand drought conditions makes this plant a good source of genomic resources for plant improvement. We identified 21 HSFs in the genome of *M. oleifera* (MolHSF1-MolHSF21) ranging in length from 110 to 1,530 amino acids. All of the MolHSFs were predicted to contain either NLS or NES or both (Table 3).

**TABLE 3:** Properties of HSFs identified in *M. oleifera*.

Gene ID	Length	Stability	pI	NLS	NES
MolHSF1	286	Unstable	6.9	P	P
MolHSF2	411	Unstable	5.67	P	P
MolHSF3	1,530	Unstable	Undetermined	P	–
MolHSF4	575	Unstable	4.95	P	P
MolHSF5	233	Unstable	8.31	P	P
MolHSF6	277	Unstable	4.95	–	P
MolHSF7	320	Unstable	5.14	–	P
MolHSF8	291	Unstable	5.96	P	–
MolHSF9	367	Unstable	Undetermined	–	P
MolHSF10	363	Unstable	4.63	–	P
MolHSF11	110	Stable	5.12	–	P
MolHSF12	472	Unstable	5.08	P (low score)	
MolHSF13	144	Unstable	5.72	Most probably cytoplasmic and nucleus	P
MolHSF14	398	Unstable	4.89	P	P
MolHSF15	650	Unstable	9.33	P	–
MolHSF16	325	Unstable	5.66	P	–
MolHSF17	679	Unstable	5.69	P	–
MolHSF18	445	Unstable	5.42	P	P
MolHSF19	362	Unstable	4.95	P	P
MolHSF20	397	Unstable	5.84	P	P
MolHSF21	623	Unstable	5.25	P	P

### Phylogenetic Analysis and Classification

**FIGURE 4:**
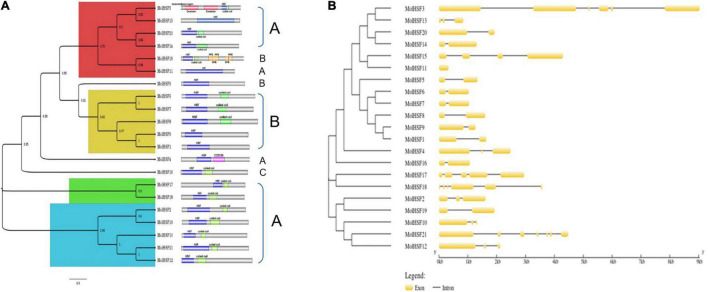
Phylogenetic analysis and classification of MolHSFs. **(A)** Classification into three classes of HSFs based on homology with AtHSFs. **(B)** Gene structure of MolHSFs.

**FIGURE 5:**
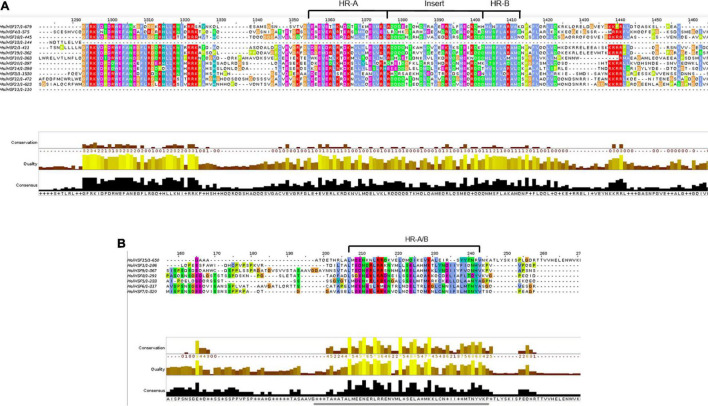
**(A)** Members of Class A MolHSFs. It comprised HR-A and HR-B motifs separated by an insert. **(B)** Members of Class B MolHSFs. It contained a HR-A/B motif with no discernible insert between the two.

**FIGURE 6:**
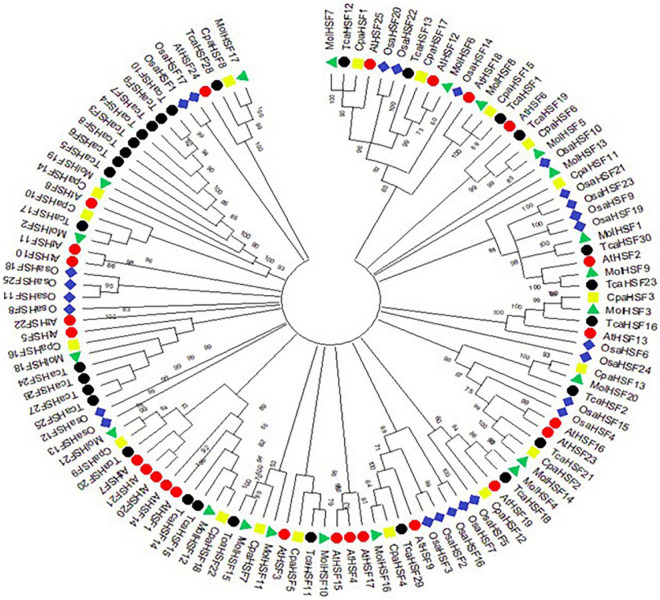
Molecular phylogenetic analysis using maximum-likelihood method and JTT matrix-based model. MolHSFs displayed closer homology to the HSFs from *C. papaya* and *T. cacao*, while *O. sativa* was a clear outlier.

The protein sequences of MolHSFs were grouped according to the homology between them. The phylogenetic tree thus generated reflects the classification of the MolHSFs into three classes, namely, A, B, and C (Figure 4A). These similarities were also reflected in the gene structures of the *MolHSFs* where members of a clade displayed similar intron–exon composition (Figure 4B). We aligned the members of Class A and Class B, respectively, and found the distinct features typical to the classes. The members of Class A are comprised of HR-A and HR-B motifs separated by an insert (Figure 5A), while the members of Class B contained a HR-A/B motif with no discernible insert between the two (Figure 5B). We then compared the protein sequences for HSFs from four other plant species, namely, *A. thaliana*, *O. sativa*, *C. papaya*, and *T. cacao*, with the MolHSFs. The consequent phylogenetic tree revealed that MolHSFs displayed closer homology to the Hsfs from *C. papaya* and *T. cacao*, while *O. sativa* was a clear outlier (Figure 6). These results are similar to those obtained from orthologous groups formed by comparing the protein sequences of various plants in this study.

### Conserved Motifs and Duplication Events in MolHSFs

The protein sequences of all MolHSFs were aligned to reveal the highly conserved regions of the HSFs (Supplementary Figure 3), and the most conserved motifs were “PFL/W/FKH/NFSSF/RQLN/GF/WEF.” Analysis of duplication events revealed the presence of two pairs of tandemly duplicated HSFs, *MolHSF17/MolHSF18* and *MolHSF19/MolHSF20*. The Ka/Ks ratios for these pairs were calculated to be 0.203 and 0.168, respectively, which suggests purifying selection (Supplementary Table 10).

### Abiotic Stress-Associated Motifs in Promoter Region of MolHSFs

The conserved motifs present in the promoter regions can provide useful speculations regarding the functional role of a gene. We identified a number of motifs associated with abiotic stress response in plants in the promoter regions of *MolHSF* genes (Table 4). The presence of such motifs indicates that *MolHSFs* have a role in the plant’s response to abiotic stress.

**TABLE 4:** Identification of conserved motifs in promoter region of MolHSFs.

Type of Motif	Description	Motif sequence
ABRE	cis-acting element involved in the abscisic acid responsiveness	TACGTG; ACGTG; CACGTA; CACGTG; CGCACGTGTC; CGTACGTGCA; AACCCGG
STRE	cis-regulatory element able to mediate transcriptional induction by different forms of stress	AGGGG
LTR	cis-acting element involved in low-temperature responsiveness	CCGAAA
TC-rich repeats	cis-acting element involved in defense and stress responsiveness	ATTCTCTAAC;GTTTTCTTAC
MBS	MYB binding site involved in drought inducibility	CAACTG
DRE core	cis-acting regulatory element involved in cold and dehydration response	GCCGAC
MYB	cis-acting element involved in drought responsiveness	TAACCA; CAACCA; CAACAG; CAACTG; TAACTG; TAACCA

### Quantitative Expression of MolHSFs Under Conditions of Drought Stress

**FIGURE 7:**
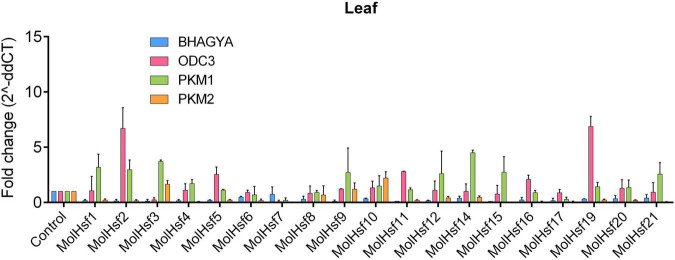
RT-PCR expression analysis of 19 MolHSF genes in leaf tissues of Bhagya, ODC3, PKM1, and PKM2 varieties of *M. oliefera.*

**FIGURE 8:**
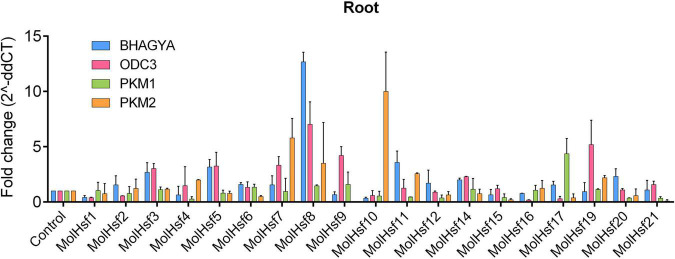
RT-PCR expression analysis of 19 MolHSF genes in root tissues of Bhagya, ODC3, PKM1, and PKM2 varieties of *M. oliefera.*

We selected four varieties of *M. oleifera*, *viz.*, Bhagya, ODC3, PKM1, and PKM2, and compared the expression of the candidate HSF genes in leaf and root tissues of these varieties. The qPCR analysis showed that HSF genes designated as MolHSF-2 and MolHSF-19 showed a significant upregulation in ODC3 leaf tissues with a fold change of more than 5 in response to drought stress, while in PKM,1 var. MolHSF14 showed a fold change of >4 (Figure 7). In root tissues, MolHSF-8 was highly upregulated in Bhagya as compared to control, while in PKM2, MolHSF-7 and MolHSF-10 showed an upregulation of >5. MolHSF-8 was upregulated in root tissues of all four varieties of *M. oliefera* (Figure 8). Other than PKM1 leaf tissues, a downregulated trend was observed in six HSF genes of Bhagya, ODC3, and PKM2. Out of the 21 identified HSFs, 19 HSFs showed gene expression in leaf and root tissues of all four varieties, except MolHSF13 and MolHSF18. Moreover, the qPCR results showed that there was good consistency between the expression levels of the genes analyzed by qRT-PCR and their levels detected using RNA-seq. Consequently, the qPCR analysis results confirmed that the data we obtained from RNA-seq are reliable (Supplementary Figure 4).

## Discussion

Good-quality genome assemblies of important plant species have become a necessity for mining valuable genomic resources for genetic enhancement and crop improvement. Contemporary methods have made it possible to generate more complete genomes in shorter time. Therefore, “genomics” and related studies have been able to provide valuable resources to facilitate crop improvement. *M. oleifera* is an important plant species known widely for its nutritive and immunity-boosting value. The plant is also drought tolerant. We assembled the genome of *M. oleifera* var. Bhagya using long reads (PacBio) and short reads (Illumina) to generate better genome coverage. The final assembly of 915 contigs was tested for quality using referenced standards like BUSCO (Simão et al., 2015), LAI (Ou et al., 2018), and N50 value and was found to be superior in quality to previously reported genome assemblies of *Moringa* (Tian et al., 2015; Chang et al., 2018). It was interesting to note that the genes predicted in *M. oleifera*, a member of the order Brassicales, had higher similarity to those from *T. cacao*, which belongs to the order Malvales. A more comprehensive analysis of orthologous groups suggested that *C. papaya* and *T. cacao* share greater homology with *M. oleifera*. Single-copy orthologs are genes that are essential to plant growth and function given their conservation in various species. Therefore, we analyzed the single-copy orthologs identified by comparing protein sequences from 11 other plant species along with *M. oleifera*. The list of contained proteins such as WRKY and zinc finger-containing and HSFs. The role of WRKY and zinc finger-containing transcription factors in abiotic stress response has been very well documented in various plants (Khan et al., 2018; Yoon et al., 2020). Both of these are classically large TF families comprising many members, which makes their role in plant development more diverse and not limited to stress response (Li M. et al., 2020; Lyu et al., 2020; Wani et al., 2021). More recently, the focus has shifted to smaller families of transcription factors, which have a more direct role in abiotic stress response in plants.

Heat stress transcription factors in plants regulate the expression of HSPs and thereby mediate the plants’ response to abiotic stress (Wang et al., 2017). Despite what their name suggests, in addition to heat stress response, HSFs are also known to regulate response to other stresses such as cold, salinity, and drought (Li Y. Z. et al., 2020). They are a relatively small family of TFs with 22 and 25 members reported in *Arabidopsis* and rice, respectively (Guo et al., 2008), 38 HSFs in soybean (Li et al., 2014), 16 and 17 HSFs, respectively, in two species of wild peanuts (Wang et al., 2017), 33 HSFs in radish (Tang et al., 2019), and 32 HSFs in lettuce (Kim et al., 2021). We identified 21 putative HSFs in the genome of *M. oleifera* and divided them into three classes based on the structure of the HR-A/B domain that is responsible for the protein interaction activity of HSFs (Scharf et al., 2012). Comparison with HSFs from other plants revealed that MolHSFs are phylogenetically more similar to HSFs from *C. papaya* and *T. cacao* as compared to *A. thaliana* and *O. sativa*. These results are similar to those obtained in the analysis of orthologous groups and point toward the homogeneity of evolution of the HSF gene family with respect to the genome of *M. oleifera*. Also, the Ka/Ks ratio of less than 1 for the two pairs of tandemly duplicated MolHSFs suggests purifying selection, pointing to the conserved nature of these TFs over the course of evolution. This makes HSFs an essential part of plants’ development and survival.

Many transcription factor families have been documented in their ability to regulate plants’ response to abiotic stress. Most of these studies have also reported a number of motifs associated with stress response that are present in the promoter regions of these transcription factors. We analyzed the promoter sequences of the *MolHSF* genes and found a number of such motifs like “ABRE,” “STRE,” “LTR,” “MYB” etc. to be present. To further study the involvement of *MolHSFs* in drought stress response, we analyzed the expression of *MolHSFs* in the young plants of *Moringa* subjected to drought stress.

Basal-level expression of 19 MolHSFs was seen in Bhagya and PKM2 var. in leaf tissues. While in PKM1, most MolHSFs were upregulated, in ODC3 var. MolHSF-2,-19 were significantly upregulated. Most MolHSFs showed a higher expression in root tissue as compared to the leaf, confirming the expectations that root is the first tissue that senses and is affected by drought stress and roots respond faster to stress than leaves, undergoing more complex gene regulation during water deprivation. MolHSF-8 presented high expression in all the four varieties of *Moringa oliefera*, suggesting an important regulatory role during drought stress. It was interesting to note that a contrasting trend was observed in the expression of MolHSFs of Bhagya and PKM1. While basal-level expression of most MolHSFs was seen in the leaf tissues of Bhagya, a high expression trend was observed in PKM1 leaf. Conversely, the trend was reversed in the root of Bhagya and PKM1 variety.

## Conclusion

*Moringa oleifera* is an important multipurpose plant with medicinal and nutritional properties and with the ability to tolerate drought, which makes it an ideal candidate to study the regulatory mechanisms that modulate drought tolerance and its possible use in agroforestry system. We carried out WGS of this species and assembled about 90% of the genome of *M. oleifera* var. Bhagya into 915 scaffolds with a N50 value of 4.7Mb and predicted 32,062 putative protein-coding genes. The 21 HSFs identified in *M. oleifera* were phylogenetically more similar to HSFs from *C. papaya* and *T. cacao* as compared to *A. thaliana* and *O. sativa*. Also, the Ka/Ks ratio of less than 1 for the two pairs of tandemly duplicated MolHSFs suggests purifying selection, pointing to the conserved nature of these TFs over the course of evolution. This makes HSFs an essential part of plants’ development and survival. qRT-PCR showed that *MolHSF8* could be a promising candidate for functional characterization in drought tolerance in plants. Further characterization of identified HSFs in *M. oleifera* and their functional validation in a panel of genotypes under varying abiotic stress conditions will help in divulging new sources of stress-resistant genes for improvement of this miracle plant.

## Data Availability Statement

The original contributions presented in the study are publicly available. This data can be found here: National Center for Biotechnology Information (NCBI) BioProject database under accession numbers PRJNA394193, PRJNA765946, PRJNA747889, and PRJNA756620.

## Author Contributions

PSS contributed to the sample collection, experimental data collection, and visualization. SP was responsible for bioinformatics data analysis and equally contributed with PSS in experimental designing. AP and SP supervised the research work. AP, SP, PSS, and MP wrote and edited the manuscript. All authors contributed to the article and approved the submitted version.

## Conflict of Interest

The authors declare that the research was conducted in the absence of any commercial or financial relationships that could be construed as a potential conflict of interest.

## Publisher’s Note

All claims expressed in this article are solely those of the authors and do not necessarily represent those of their affiliated organizations, or those of the publisher, the editors and the reviewers. Any product that may be evaluated in this article, or claim that may be made by its manufacturer, is not guaranteed or endorsed by the publisher.

## Footnotes

1
https://www.bioinformatics.babraham.ac.uk/projects/fastqc/

2
https://www.aggenome.com/

3
http://ftp.ebi.ac.uk/pub/databases/GO/goa/

4
http://ftp.ebi.ac.uk/pub/databases/GO/goa/goslim/goaslim.map

5
http://www.repeatmasker.org/RepeatModeler/

6
https://tandem.bu.edu/trf/trf.html

7
https://phytozome.jgi.doe.gov/

8
busco.ezlab.org

9
https://github.com/TransDecoder/TransDecoder

10
https://github.com/rambaut/figtree/releases

11
https://github.com/deweylab/RSEM

12
http://bioconductor.org/packages/release/bioc/html/edgeR.html

13
http://kobas.cbi.pku.edu.cn/

14
http://bioinfo.cau.edu.cn/agriGO/

15
http://planttfdb.gao-lab.org/

16
https://ftp.ncbi.nlm.nih.gov/blast/executables/blast+/LATEST/

17
https://bcf.isb-sib.ch/Delorenzi/Marcoil/index.html

18
http://www.moseslab.csb.utoronto.ca/NLStradamus/

19
http://nls-mapper.iab.keio.ac.jp/cgi-bin/NLS_Mapper_form.cgi

20
http://www.cbs.dtu.dk/services/NetNES/

21
http://dog.biocuckoo.org/

22
http://meme-suite.org/

23
http://gsds.gao-lab.org/

24
http://www.jalview.org/

25
http://bioinformatics.psb.ugent.be/webtools/plantcare/html/

26
https://www.uniprot.org/uniprot/?query=reviewed:yes

27
http://ftp.ebi.ac.uk/pub/databases/Pfam/current_release/Pfam-A.fasta.gz

28
https://www.genome.jp/kegg/kaas/

29
https://ftp.ncbi.nih.gov/pub/COG/COG2020/data/

## Supplementary Material

Supplementary Figure 1 *Moringa* varieties, *viz.*, Bhagya, ODC3, PKM1, and PKM2, during drought stress treatment.

Supplementary Figure 2 Gene enrichment analysis for DEGs in response to drought stress in *M. oleifera*. **(A)** Enrichment of genes in KEGG pathways and **(B)** enrichment of GO terms in the DEGs.

Supplementary Figure 3 Conserved motifs of MolHSFs. The most conserved motifs were identified after alignment and visualized with MEME suite.

Supplementary Figure 4 **(A)** Graph depicting the trend of pattern of expression observed *in silico* (RNA-Seq) and with qRT PCR. **(B)** Scatter plot for the correlation between log2 values of fold change observed in digital expression analysis for RNA-Seq and validation with qRT PCR.
