# Supplementary material for: De novo Whole-Genome Assembly of Moringa oleifera Helps Identify Genes Regulating Drought Stress Tolerance
Source: Front Plant Sci. 2021 Dec 14;12:766999. doi: 10.3389/fpls.2021.766999 (PMC8712769; doi:10.3389/fpls.2021.766999)
Supplement: Supplementary Figure 1 — Moringa varieties, viz., Bhagya, ODC3, PKM1, and PKM2, during drought stress treatment. [file Data_Sheet_1.zip › Supplementary Data 1.PDF]

Supplementary data S1: Sequences of HSFs identified from M.oleifera var Bhagya genome.

CDS sequences
>MolHSF2
ATGAATCCTTACGGTAGACTGGATGAAGAGTACCCAGGAGCTAGTTCTCCGTATTCTGGCATGCCTTCTATAGCAACTC
CACAACCAATCGAGGGTCTCTACGATGCAGGCCCCCACCTTTTCTTAGAAAAACGTACGATATCGTTGATGATTCAAGCACT
GATCATATAGTTTCT
TGGAGCAGAGGCAACAATAGCTTCGTCGTGTGGGATCCCCAGATCTTTTCAAGGAGTCTTCTCCCTAAATATTTCAAGCACAA
CAATTTCTCTAGCTT
TGTAAGGCAGCTTAACACCTATGTGAGGAAATGGACAAGGGCTGTTATCAGGCCTTGCTCTGCTCTAGCATCTGCACTCCAGT
GCTGTACCATTAGCG
TTGTTAAAGCTTTCATGAATATTAACTTTGATGTGTTCACTTCTAATCCTTTATTGCTTTTGAATGGGTTCAGGAAAGTTGAT
CCAGATAGATGGGAG
TTTGCTAATGAAGGTTTTCTGAGGGGACAGAGGCATCTTCTCAAGAATATAAGGAGAAGAAAGAGGACTGATATGCAGGCTTC
ACAGCAAGCTCTAGA
CCCTTGTGTTGAAGTTGGGAGGTTTGGGTTAGATGGAGAACTTGATCGGTTGAGGCGTGACAAACATGTTTTAATGTTGGAAT
TGGTGAAGCTTAGAC
AGCAACAGCAGAACACCAAAGCTCATCTTCAAGTAATGGAAGAAAAGCTAAAAAGCACAGAAATAAAACAGAAACAGATGATG
AGTTTCTTGGCAAGA
GCAATGCAGAATCCTAACTTTGTACAGCAATTAGTACAACACAAAGATAAGAGAAGAGAACTTGAGGAAGCAATTAGCAAAAA
AAGAAGGCGACCAAT
TGATCAAGGACGAAGTAACATTGTAGTTGGTGAATTAGACCAAGGTGGAAGTTCAGGAGTTTTTGTTAAGATTGAACCCAAGG
AATATGGTGACATCT
CGGAGTTTGAAGTTTCAGAGCTAGACAAACTTGCTGTGAATATGCAAGGATTAAGTGAATCCCCAAGTAACCTGGAGCAGCAG
CACACAGAGAAAAGG
GAGGAGCATGGAAGTAAATGTAAAGACATCGATGAGGAGTTCTGGGAAGACTTGTTGGATGAGGACATTCAAGAAGAAATGGG
GGTACTGGATGTTGA
AGGTGAAGCTGAGGAAGATGTAAATGTTTTGGTAGAGCAGCTGGGCTTCCTGGGTTCTAGTCCTCAGTATAGAAACTGA
>MolHSF3
ATGACTGTTGACGGGTTGATTAGAGAAAGTTATGGTATTGGAAAGCCCAGCAGGAGCAGATGGAGAAACAAGCCGCCTA
GAAAATGCAGTGGCAAACAACTACGGATTTTCATTCTTTTGTTAGTTGTTGTTTTGTTCTTTTTATTGGTTTCTGATCGTTCT
CTGTCTCCTCTTCCT
GGCAAGATTGGGTTCAGTTTTGCTGTGGGAAACTATGTACAGGACAGACCTCGGTTAGTTGCCGGAAATTTTAGCAGACGGCA
AGAGGTGAAAGCAGA
GTCTGATAAGGATTTGTGCCGGGGACGATACGTTTATGTTCATGATCTTCCAAGCAGATTCAACCAGGATGTGCTCAAGAGTT
GCCGATTGCTTAACA
GGCCGACTGATAAGTCCAACATGTGTGATAACCTTTTGAACCTTGGTTTTGGCCCTCGAATTGAGAACTCCGATGGGGTTTTA
TCAGACAGGAGTTGG
TACTCTACCAACCAGTTTCTGTTAGAGGTTATATTTCACAACAGAATGAAGAATTACGAGTGCCTGACATATGATTCTTCGCT
TGCATCGGCCATTTT
TGTGCCGTTTTATGCTGGTCTGGATCTTCGCCGCTATCTATGGCGTCTTAAAACGTCGGTCAGAGACTCATCTGGGATTGATC
TCGTGAGGTGGCTGG
CGGGGAAGCCTGAATGGAGAAGAATGGGGGGCAGGGATCATTTTCTGGTTGCAGGGAGGATTTCTCGGGATTTCAGGAGACAA
TCAGACAAGAATTCT
GATTGGGGCAGCAAGTTCAGGCTCCTGCCTGAATCCCAGAACATGTCAATGCTGTCAATTGAATCCAGCTTCTCCAAAAATGA
CTTTGCCATACCATA
CCCAACATACTTCCATCCTTCTTCGGAAAGTGAGATCTTCCGGTGGCAAGACAGGATGAGAAGAAACAGACGGGAATTCTTGT
TCTCTTTCGCCGGTG
CACCAAGATCCAGAAGGAAAGGTTCCATCAGGGAGGAGGTTATCAGCCAATGCAAAGCTTCAACCAAAACATGCAAGTTTCTG
GACTGTGATGCTGCT
ACACAGGGTTGCGAGGACCCAGTTGAACTGATGAAACTGTTTCAGAAATCAGTCTTCTGCTTGCAGCCTCCCGGGGATTCCTT
CACCAGGAGATCAAT
CTTCGATTCAATTTTGTCAGGCTGCATTCCTGTTTTCTTCCATCCAGGCAGTGCTTACGAACAATACAAATGGCACTTACCCA
GGAATCACACAGAGT
ACTCAGTGTACATACCACCCAAGCAATTGAAATCTGGGAAACATAGCATAGAGCAGACTTTACTGGGAATCAGACGGGACAGA
GTGGAGGCTTTAAGA
GAGGAGGTCGTCCGGCTGATTCCAAGAATCACATACAGAGATCCAATGTCTAAATTAGAGACCATTGAAGATGCGTTTGATTT
AGCAATCAAGGACAG

GAGCTTTCCTCCAGAATTGCAGAGTCTTCTTGCTTGTGTTTACCGTCCTGTTGCGAGGATGAGTTCTGATGGGTTGATTAGAG
AAAGCTGTGGTTTTG
GAAAGCCTGGCAGGAGCAGATGGAGAAACAAGCCACCGAGGAAATGCAGCAGCAAACAACTATGGCTTTTCATTCTTTCCTTC
GTTGTTCTTTCTTCC
TTTTTGTCGGTTTCTTATCATTCTGTGTCTCCTCTTCCTAGCAAGATTGGGTCCACGTTTTCAATGGAAAACTTCGTCnnnnn
nnnnnnnnnnnnnnn
nnnnnnnnnnnnnnnnnnGTTTGATGAGTGTTTGTGCCGGGGACGATACGTTTTTATTCATGATCTTCCAAGCAGATTCAACC
AGGACGTCCTCAAGA
ACTGCCAATGGCTTAAGAGGCCCACTGAGGAGTCCAACATGTGCAATTACATACTGAACCTTGGTTTTGGTCCTCAAATTCAG
AACTCTAACGGGGTT
TTATCAGACAGGAGTTGGTATTCTACCAACCAGTTTCTGTTAGAGGTTATTTTTCACAACAGAATGAAGAATTACGAGTGCCT
GACAAATGATTCCTC
GCTTGCGTCGGCCATTTTTGTCCCGTTTTACGCTGGCTTGGATCTTTACAGGTACCTCTGGCGGCCTAACTTATCGGTGAGAG
ATTCATCTGGGATTG
ATCTCGTGAGGTGGCTAACAGGGAAGCCTGAATGGAGAAGAATGGGAGGCAGGGATCATTTCCTGGTTGCAGGGAGGATTTCT
CGGGATTTCAGGAGA
CAATCAGACAAGCATTCTGACTGGGGCAGCAACTTCAGGCTTCTGCCTGAATCGCAGAACATGTCAATGCTGTCAATTGAATC
GAGCTTCTCCACCAA
TGACTTTGCCATACCATACCCAACGTACTTTCATCCTTCTTCAGAAAGTGAGATCTTACAGTGGCAAGACAGGATGAGAAGAA
ACAAACGGGAATTCT
TGTTCTCCTTCGCAGGTGCCCCGAGACCGAGGACGAAAGGTTCTATCAGGGAAGAAGTCATCGACCAATGCAAAGCATCAAAT
AAGACATGCAAGCAT
CTGGACTGTGACGCTGCTGCACACAGTTGCGAGCACCCAGTTGAGCTGATGAAACTGTTTCAGAAATCAGTCTTCTGCTTGCA
GCCTCCTGGGGATTC
GTTCACCAGGAGATCAATTTTTGATTCAATTTTGGCAGGCTGCATTCCTGTTTTCTTCCATCCAGGCAGCGCTTACCAGCAAT
ACAAATGGCACTTAC
CCAGGAATCACACAGAATACTCGGTGTACATACCCTCCAAGCAATTGAAATCTGGAAACCATTGCATAGAGCAGACTTTACTG
GGAATCAGGGAGGAA
ACAGTGGAGGCTTTGAGAGAGGAGGTTCTCAGGCTGATTCCAAGAATAACATACAGAGATCCAATGTCTAAATCAGAGAACCT
GGAAGATGCATTTGA
TCTAGCAATCAAGGCCTTTATATTTAATTGGGGAAAGCACATGATCGACGGCTGTTTCCTGATCATAAAAGAAGTGGAAGTTT
ACAGATCGATCCAAA
AACCCAGAAGCCGGAGCACCACTGCCCAAGTCACCTGCAGAGAGGGGAGAATGGAAGCTGTCACGGGGACATCCAGCAGTGGC
GGAGGCGGGGGAGGA
GGAGGGCCGGCGCCTTTCTTGTTGAAGACGTACGACATGGTGGATGACTCATCGACCGATGAGATCGTATCCTGGAGCTCTAA
CAACCGCAGCTTCGT
CGTCTGGAACCCTCCCGAGTTCGCGCGTCTCCTCCTCCCTACCTACTTCAAGCACAACAACTTCTCTTCCTTCATCCGCCAGC
TCAACACCTATGAAA
TTCTATTGGGTCTTTTGGAATTAGATTTTGTTGTTGATGCGTGTTATATGAGGATTGATTTCCAAGAAGTCGGATTCCGAAAG
ATTGATCCTGAGAGA
TGGGAATTTGCTAATGAGGATTTTATTAAAGATCAAAAGCATCTTCTTAAGAATATCCATCGCAGAAAACCTATTCATAGCCA
CACTCACCCTCATGG
TTCTATGGTAGATCCAGAAAGAGCAGCATTTGATGAAGAAATAGAGAGGTTATCACGTGAGAAAGCTATGCTTGAGGCAAACA
TTTTAAGGTTCAAAC
ATCAAAGGTCAGCTGAGAAGCATCAGTTGGAAGACATGACCCGCAAAATGGATAGTATGGAGCAGAAGCAGGAAAACTTGCTC
ACCTTCTTACAAAAG
GCGGTTCAGAACCCTGCTTTTGTTGAGCATCTTGCACGAAAAATTGAATCTATGGATATCTCAGCTTATAATAAGAAAAGACG
ACTTCCTCGGCCTGA
GAGCTCAAAGCCAGTTGTGGAAAATAGTTCTTTGGATAACAACAGTAGTTCTAGACCTGAGTTTGGGAACATTTTCCACCAAG
ATTTTTCAAATAAGC
TGAGATTAGAGTTATCGCCAGCAATTTCAGATATTAACTTGGTTTCACATAGTACTGAGTGTTCTAATGAAGATGGGGGAAGT
CCTCAGAGGAAAATA
TCTGAAGGAGATCCAAAAGATGCACACACAAGAACAGAAGGTCTTTCATTTGCGCCTGACATATTAGAACTTTCAGACACTGG
AACATGCCCACGAAA
AATACCATTACATGAAAGCTCCAGGGTGCATGCTTTTCAGCAGAGATTAACTTCTACTGAAGATGTTGATGGTCATATTTCCT
GTCATTTAAATCTAA
CTTTGGCATCTTCGCCATTGCCAGTCAACAAAAGCCCTTACCCAGCTAGGATGTCCCAGCACAATCAGGTAATTGGAAAATTC
CCTGACTCAAGGTTT
AATGTCAACACCAAAGAAGCTGATAACAGAGGATTTCTGAAGAACAGAAATCTGGCTGATGAGAACACGACTTTATCTTCCTC

TCGAGAGGCCCCAGA
TAACTATCAAGGGACCACAGCTCCTCCAGGTAGAGTTAATGATGTATTCTGGGAACAGTTCCTAACTGAAAGACCAGGGTCTT
CAGACAATGAAGAGG
CGAGTTCTATTTACAGGGCAAACCCATATGAGGAGCAAGAAGATAGAAGGCCAGGTCAAGGGATATCAAGAAATTCCAGGAAT
ATAGCACAGCTCACT
CTCTGA
>MolHSF19
ATGAATCCATTCTATCCCGTCAAGGAAGAGTACCCAGGTTCCAGCTCTTCGCTGTCCGCCGATGACCCGACATTGATGC
CACCGCCGCAGCCAATGGAGGGTCTTCATGACACAGGGCCGCCCCCATTCCTCACTAAGACATTCGATATGGTTGATGACCCA
AATACCAATCATATA
GTTTCTTGGAACAGAGGAGGTAGCGATTTCGTTGTGTGGGATCCTTATTCCTTTTCCACTAATCTCCTACCCAGATACTTCAA
GCACAGCAACTTCTC
CAGCTTTGTCAGGCAGCTAAATACATATGGCTTTAGAAAGATTGATCCAGATAGATGGGAATTTGCCAATGAAGGATTTTTGA
GGGGCCAGAGGCATC
TTTTGAGGAACATCAAAAGAAGAAAGGCGCCTTCTCAGCCTCTCCCTCCACAGCAAGCTGTTGGTCCTTGTGTTGAAGTTGGC
AGGTTTGGATTAGAT
GGAGAAATGGATCGTTTGAGGCGTGACAAGCAGGTCCTTATGATGGAACTAGTGAAGCTTAGGCAACAACAACAGGACACTAG
AGTTTACCTTCAGGA
AATGGAACAGAGGCTGCAATTCACAGAAAAGAAGCAACAACAAATGATGTCCTTCTTAGCTAGAGCAATGCAAAATCCAGCTT
TTTTGCAGCAACTAG
CTCAACAGAAGGAGAAGAGATTGGAGCTTGAAGAAGTGATGACGAAGAAAAGGAGGAGGCCAATTGATCAAGGACCTGGGCGT
GTTGGCGAAGGTGAA
TCAAGTAGGAGTGGGGAGGGAGTGAATTCCGTTAAGAGTGAACCTCTTGAATATGGTCTTGAAGTGTCAGAGCTGGAAGCACT
TGCACTGGAAATGCA
AGGGTATGGTAGGGCAAGAAAGGAGCAAGTGGAGGAGCAAGAAGAGCTGGAGCTGGAGCCAGGGGAGGGAGGAGACAGAGAAC
TTGACGAGGGATTTT
GGGAGGAGTTACTGAGTGATGAGAGATTTGAGGGAGAACTGGATATCCCAGGTGCAGAAGGAGGTGAACATGAAGATGTCAGT
GTATTGGCTGACCGT
CTGGGTTATTTGGGTTCTAGTCCAAAATAG
>MolHSF20
ATGGATAACTCCCAGGGTAATTCAAGTGCACCACCACCTTTTCTTTTAAAGACGTATGAGATGGTGGATGATCCAGTGA
CAAACTCCATGGTGTCTTGGAGTCCAAGTGGTTGCAGTTTCATTGTGTGGAATCCACCAGATTTTGCCAGAGACTTGCTGCCC
AAGTACTTCAAGCAT
AACAATTTTTCAAGTTTTGTGAGGCAGCTTAATACTTATGGTTTCAGGAAGATCGATCCTGATCAGTGGGAGTTTGCCAATGA
TGAGTTTATAAGAGG
AGAGAGACATCTTCTAAAGAATATCCACCGAAGAAAGCCAATTCATAGCCATTCTGCTCAGCTTCGGGATGGTTCCTCTCTTC
AATTAGCTGAATCAG
AAAAGGAGGAATTTCAAAAGGAAATTGAGCAACTGAAACATGACAAGAGCTTGCTTTATTTGGAGCTACAAAAGCGTCAGAGA
GAAATCCAAGGGTTT
AAGTTACAAAGACGATCATTACATGATCGTTTGCAGAACATGGAACATAGGCAGATGAAGTTAACATCCTTCTTGGCTCAACT
TTTGCAGAAACCAGG
CTTCGCGTCTATGCTTTTGCAACAATCACAAATTCATACCAAAAAGAGAAGGTTATTGAAAGTTGATCATTCAAATGATGAAT
CTAATGTATTAGAAG
ACTGGAAATTGATTGTCCAGAAAGAAAACATGGATGAAAGTACTGCCCTTATATTGGATTCTCAACGGGTTGAGAAGCTGGAC
TCATCCTTAAAATTC
TGGGAGAGCATTTTGCATGGGGCTGGTGAAGCTTTTGGTGAAGTGCATGATTCTAGTAGGTTGTCTTACCCCTCACGAGTCCT
CGTCACAGAACTGCA
TGCATCCTCAGAAGATCATGATGTGCACGTAGAACCTAGCTCACCTAGAGTGCCTTCATCCTCTTGCCAGTTAAGGAACGTTC
ATTCTTCTCCAGAGT
TAGCTGGCTCTATGTATCATGTTGGGAGTCCTGTATCTCATCTTACAGCTGACATGAGGCCAAAAACTTTGGAGATGGATGTA
AACCCTGAGCTCACA
AGTGCTTCAAATGCAGAGGCTTTAGATGAATATACATCAGGTACAACAAATCCTTCTGCATCGACGAGGGTGAATGATCTCTT
CTGGGAACAATTCCT
AACTGAGACCCCTGGTTCATTTGCTGAACAGGAATGA
>MolHSF9
ATGGCTCTCATGCTAGAAAACTGTGAGGGCATATTACTGTCACTGGACTCTCACAAATCTGTGCCAGCGCCATTCCTCA
CTAAAACGTATCAACTGGTCGATGATCCTGCCACCGACCACATAGTCTCTTGGGGTGAAGACGACACCACCTTTGTTGTATGG
CGCCCGCCTGAATTC
GCTCGCGACCTTCTTCCCAACTACTTCAAGCACAATAACTTCTCCAGCTTCGTCCGCCAGCTCAACACCTATGGTTTTAGGAA

GATTGTACCAGACAG
ATGGGAGTTCGCCAACGAGTTCTTCAAGAAGGGAGAGAAGCACTTGCTCTGTGAGATCCACAGAAGGAAAACAGCGCAGCCAC
AAGTAGCCATCAATn
nnnnnnnnnnnnnnnnnnnnCGCATTCTCCACTCGGTGTTAATGGACCCGGTTTCTTCCCGTTTCCTAGCCGAGTCAGCATA
TCGCCGTCTGACTCG
GACGAGCAAGCCAACTGGTGTGACTCGCCCCCACTGTCTTCTCCCAGAGGAGCCACTGGGGTCTCAGTTGTCAGTACGGCTTC
TGCAGCTGTTGGTGG
TGCTTATAACAACTCAGTCACAGCTTTGACAGAAGATAACGAGAGATTAAGAAGAAGCAACAATATGCTCATGTCCGAGCTCG
CACACATGAAAAAAC
TCTACAATGATATCATTTATTTTGTTCAGAACCATGTCAAGCCAGTGGCTCCGAGCAATTCTTACCCTTCCTCTTTGCTCCTT
TGTAACCCACCACCG
TCTGCTACTACGCCTCTAGGTACCACTAACTGTTCGTTGGGCCAAAAGCCCTTGAACCAGCTTCTTGGATACTACCCTACTAA
TCCGAAACAAGCCCC
TCAAGTTCAGGTTTTGAACTCTCCAACGACCACAGCGCAGAGCACTGTGACGATCGTCGAAGAGGCTAAAAACAATAGCTGCA
AGACGAAGCTTTTTG
GTGTGCCTCTACAATCGAAGAAGAGATTGCACCCAGAATACGGTTCTGGTCAGGCAACGATGGAAACCAACAAAGCTCGTTCT
CGTTTGGTGTTGGAA
AAGGATGATTTAGGGTTGAATCTTATGCCTCCTTCAACATGTTAG
>MolHSF1
ATGGCGTTCACTGTAGAGAGATGTGAGGAGATGGTGTTCTCTGTAGAGTCTCAGAAGACAGTGCCTGCGCCGTTCCTGA
CCAAGACGTATCAGCTTGTGGATGACTCTCTTACCGACCACATTGTGTCTTGGGGTGAAGATGGCACTACTTTTGTTGTCTGG
AGACCTCCTGAGTTC
GCTAGAGATCTTCTCCCCAACTACTTCAAGCACAACAACTTCTCCAGCTTCGTCAGGCAGCTCAACACCTATGGATTCAAGAA
AGTGGTTGCAGATAG
ATGGGAGTTTGGGAACGAATGTTTTAGAAAAGGAGCAAAGCATTTATTATCAGAGATCCATAGAAGAAAAACTCCTCAGCATC
ATCACCAACACTACC
ATGATCAGCAGCAGCAACCCCCGTTTCTGCAACCAGAAGAAAGCTTTGCTTGGATTGATCACTGTCCAGTGCCTTCACCAAAA
GTAAGAACTGATATT
CTCACAGCACTCACAGAAGACAACCAACGCCTGCGACGAAAGAACTATATGCTGTTGTCTGAACTGGCTCACATGAAGACTCT
TTACAATGACATTAT
CTACTTCATTCAAAACCATGTAAAACCAGTCGTTCCCCAGGAGCCAATAAGAGCCAATAGTGCTTCAGTACCTAAGCTCGTTG
AACTTGGTTCTTCAG
TGCTTCAAGATGAAACCCTTTTGGGCATTCAAGCTGTTAACAATGGCAGTATGGGTAAGCCATCGGGGGGTTCAACTGAGGAG
AGAAGTAGTATTGGT
ACTGTGAAGCTCTTTGGGGTCCCTCTCAGTGGCAGAAAGAGATTGCACTCGGAAAAGATTGATCAGCAAGAACTGGAATGTGG
GGTCATCCCATGA
>MolHSF14
ATGGATGACAATCAGGGCAGTTCAAACTCACTGCCTCCTTTCCTTACAAAGACGTATGAGATGGTGGATGATCCTTCCA
CAGATTCAATTGTCTCCTGGAGTCAGAGCAATAAAAGCTTCATTGTATGGAATCCACCTGAGTTTGCCAGTGACTTACTTCCC
AGATTCTTTAAGCAC
AATAACTTCTCTAGCTTTATCAGACAGCTGAATACATATGGTTTTAGAAAAATTGATCCAGAACAATGGGAATTTGCAAATGA
GGAGTTTATAAGAGG
TCAGCCCCAACTTCTGAAAAACATTCATAGGCGCAAGCCAGTTCATAGCCACTCCTTGCAGAATCTCCAAGGACAGGCAACAT
TGACTGAATCAGAAA
GACAGAGTTTAAAGGGTGAGATTGAAAGACTAAGGAAGGATAAGGAATTACTTCTTTTGGAGTCACTAAGGCATGAAGAGGAG
CAGCAGGGACTTGAG
TTACAAATGCAACTCTTGAAGGAGCGCCTGCAACATATGGAAAAACGTCAGCAAACAATGGTATCTCTCGTGGCCCGAGTGTT
GCAGAAACCGGCACT
TGCCTTAACTCTTTCACCACGATTGGAAACTAATGATAGAAAGAGGAGGTTGTCAAGAATTGGTTACTTGTATGATGAAGACA
CTCAGATGGGGACTT
CCCAAATTGTAGCTAGAGAAAATGGGGATAATTCATCTGCTGTGTCATCAAACACAGAGCTGTTTGAGCAGTTAGAGTCATCT
CTTGTATTTTGGGAG
AATGTTATACATGATGTGGATGAAACTTGCATTCAACTCAATGCAAACCTGGAGGTGGATGAATCTACAAGTTGTGCAGAAAG
CCCAGCTATATCTTG
CATACAACTGAATGTTGATGCCAGGCCCAAGTCACCAGAAATTGACATGAATTCTGAGCCTGCTGCTGTTGTTGTGCCTGAGC
CAGTAGCTCCTAAGG
AACAACAAATGGGGACTGCTGCTGCTCCACCAACTGGGGTCAATGATGTATTCTGGGAACAATTTTTGACTGAAAATCCTGGT
TCAACTGACACACAG
GAAGTTCAGTCTGAAAGAAAGGATCCTGATGGGAGGAGCGACAGTAAACCTGGTGATGGAAAATTTTGCTGGAACCTGAGGAA

TGTAAATAACCTTGC
AGAACAGATGGAGCATCTGACTCCTGCAGAGAGAACTTGA
>MolHSF15
ATGCCAAGAAAGTTCCTGAAGCTTAAAAAAAGTGAGATGACTTCCTCTCCAGGAACAAAGGGCCCGACTCCATTCCTGT
GGAAGACGTACGACTTGTTAGAAGAAGTTGATCGATCGTGTTGTGGAGGCAAGAGAATCGTTTCGTGGAATGGAGACGGAACT
GGGTTTGTCGTCTGG
TCTCCGGCCGAGTTCTCGGAGCTCGTGCTGCCTAAGTACTTCAAGCACAATAACTTCTCCAGCTTCGTCCGCCAGCTTAATAC
CTATGGATTCAAGAA
AATATCCATGGAGAGGTGGGAGTTCAAGCACGAGAAGTTTCAGAGGGGCTGCAAGCACAAGCTGGTGGAGATCACAAGGAAGA
AGAAGGAACTGAGTG
TGTTTCCGGCGTACCTAAAGGCTTCTTTGGACGATGCAGCAGCTGCCACACAGGAGACTCATCGGCTAGCTCTCATGGAGGAG
AACAAGAACCTAAGA
AGACAAAAGGTGGAGTTGCAGATGCAAATAAAAGAGGTTAAAGCCTTGGAGATAAGATCTTACTACACAAACAGGGTGAACAA
AGCGACCTTGTACTC
CAAAATCAGCCCATTGGGGGACCGCACTACTGTTGTACATGAGCTGGAAAATTGGGTCAAAAATGGGAACAAGGTCCGGGTCG
CTGAGCTTCAACGTA
TCATTCACGATCTTCGCAAGCGAAAGCGGTTCACTCAGGCTCTTGAGGTTTCTCTGTGGATGAATGAAAAGGGTATCTGTACC
TTCTCCCCAAGTGAA
CACGCTGTGCAATTGGATCTGATTGGTAGAGTTCATGGATTTGTTTCTGCAGAAAGCTATTTTGATAACTTGAGGGACGAGGA
TAAAACTGATAAGAC
ATATGGTGCTCTTCTGAATTGTTATGTCCGACAGCGGCAAACTGATAAGTCACTTTCCCATTTGGAGAAAATGAAGAAGATGG
GCCTTGCCTCATCAC
CCCTTACTTACAATGATATCATGTGCCTTTACACAAATATTGGCCAGCATGACAAAGTGCCTGGTGTGCTAGCTGAAATGAAG
GAGAATAAAGTCTCC
CCTGACAACTTCAGCTATAGAATCTGCATCAACTCCTATGGTGTGAGATCCGATTTAGAGGGAATGGAAAGACTTTTACAAGA
GATGGAGAGCCAATC
CAATATTGTCATGGACTGGAACACATATGCTGTTGCTGCCAACTTCTACATAAAAGCAGGCCTGAAAGATAAGGCTATTGTTC
TACTGAAAAAAATCAG
AAGAGAGATTAGAGAAGAAAGATGGTGTTGGGTATAATCATCTGATATCACTCTATGCAAGTTTGGGGAATAAGGCTGAGGTC
TTGAGACTGTGGCAT
TTGGAGAAAAGTGCTTGTAAGAGATATATAAACAGGGACTATATTACCATGCTGCAGTCTCTGGTGAAGCTTGGTGAGGTTGA
AGAAGCTGAAATAAT
ATTGAGGGAATGGGAATCATCTGGTAATTGTTACGATTTTAGGGTGCCAAACACTGTCATTATTGGGTGTGCTGAAAAAGGTT
TGCTTGACAAGTCAA
AAGCAATGCTTCAAGACTTGACAAAGAAAGGAATGACTGTTACCCCCAATAGTTGGGGTGTTGTGGCAGCAGGTTACATGAAG
AAAGGTCAGCTGGAG
AAAGCTTTTCAGAGTATGAAGGCTGCCCTCTCTGTGCATTTGGAGTTTAAAGGATGGAAACCCAACCTTAATGTGATTACAAG
CATTTTAAATTGGCT
GGGTGATAAAGGTAGCGTTGGAGATTCAGAAGCTTTTGTGGCCTTGTTGAGGACTGCAATTCCAGTAAACAGACAAATGTACC
ACACCCTGATTAAGG
CAAGTTTAAGGGATGATAGAGAAGTAGATGGGATTTTGAAACGCATGAAAGCTCATATGATAGATGAAGATGATGAAACAAAG
AAAATACTTGGCACA
AGGCAGAAGTAA
>MolHSF21
ATGGACTCTGTTCACGATTCTATATCGATAGGAAACTATGTGGAGGCAAATGGGAGTTCTGTACCGCCATTTCTGAGCA
AAACCTATGACATGGTGGATGACCCATCAACAGATTCAGTCGTGTCCTGGAGTAACAGCAACAACAGCTTCGTGGTGTGGGAT
GTGCCTGAGTTTGCA
AGGGACCTTTTGCCTAAATATTTCAAGCACAGTAACTTCTCCAGCTTTGTCAGGCAGTTGAACACTTATGTGGAGTGCATCGA
CACTGAGTTGATTCT
GTCTATAGATCTTGATTTTTCTGTTCCTATGGAGTATTTGAATGATGGGAGGCTGATTGGTTATAGATTTGAAGTTAGTTACA
TTATGATCAAAAAAA
TTTTGAGTGTCTGGTTGACTGGACTTAATTCTGAAATTGATCAGATGGAAGCTATCCCTTTGAGCATTGCTTCTTTGGCTCGT
CTGTCAGAACAAGTT
TTGCCTCTGATGCCATCTATTGCTGTGGCTTTGACTGTAGCATCCACTGTTTACTACTCTTCTGCTATCCATGTCTCATTCGA
TACATGGAACTTGAG
GTCAGGAGCTTACACTGAACTTATTTTGGAGATCATAAATTACATGCATAGGAGTGCCTTGTTTCTATCAGGAAGCATAGCCT
TGCAGTGTAGATTTT
GGATGGGTTTTAGGAAGGTTGATCCAGATCGCTGGGAATTTGCAAATGAGGGATTTCTAAGAGGTCTGAAGCACCTTTTAAAA
AGTATTGTTAGGCGA

AAACCTGCTCATGTTCATAATCATCATCAGCCTCAAGTTCAGAATACTTCTGTTGGTGCATGTGTTGAGGTTGGGAAGTTTGG
GCTTGAGGAAGAGGT
GGAAAGACTTAAGCGAGACAAGAATGTGCTTATGCAGGAACTTGTCAGGTTGAGGCAACAGCAGCAAACTACGGAACAACAGT
TGCAAACTGTTGGGC
AAAAAGTACAGGTAATGGAACAGAGACAGCAACAGATGATGTCATTTCTTGCAAAGGCCATGCAGAGTCCCGGTTTCTTAAAC
CAGCTAGTGCAGCAT
CAGAATGATAGTAATAGGCGCATTGCTGGGGGCAACAAAAAGAGGAGACTCCCCAGACAGGGCGAGGAAAATTTAGCTGGTGA
GCATCCTGCCAGTGG
TCCAAATGGGCAGATAGTTAAGTTCCAGCCTTCAATGGATGAAGCAGCAAAGGCAATGCTGCATCAAATCATGAAGATGAATG
CATCACCTAGGCTTG
AAGCATCAGTCAACAACCCTGGCACTTTTCTTATTAATGATATTTCTTCTTCCAATGTGTTAGAGAATGGAAGCTCCTTAAAT
GGTGTTTCAGGAGTG
ACACTTTCAGAAGTTTCACCAGTTTCTGGGCAATCTTCTATATCAGCAAAGTTGGGATTTCCTGTCAGTGGCACATCCACCGC
AATTACTGAGATGCA
GTCATCCCCTTGCATGGTATCTGAGAGTGGTAAAGTGGATCAAATTCCTGAAATGAATATGCATCATTGTGGAGAAGACATAA
TTTTGGCAAACTCAT
CTCAAGTGCAAGAGAATATGTCAGGAAACACTGTTGGAAACACTGATGCCAATTTCATGGATTCTGAGGTTAATGCTGTCTCG
ATTTTGGATGAGAAA
AAGGCCATAAAAACTAAGATCGTCTCTCCTGAACATGATATAGACTTCTTTATGGATGGGGCCCCCAAGCTTCCTGGAATAAA
TGATCTTTTCTGGGA
GCAGTTTCTTGCAGTGAGCCAACTGACTGTGGACACAGGTGAAATCAATTCAAGCTCCCTGGATAGTTGTGTGACCAAGGAAT
ATGAATTGCAATTGG
ATCAGAAGTATGGGTGGGACAAAATGTAG
>MolHSF12
ATGCTGCGTGCAGTCTCATGTAAGGAAATTGAGACGCCTAGCAAAATGCCTAAGAAATCTTGCATATGTTGGGTTAAAG
ATGTTTTAAGTTGGGCGCTTTCTGTTAGGTTCACTGCTTTTCAAGATTTCATGTGTTGGAGATTGTGGGAGGGTTTTAGAAAG
GTTGACCCTGACCGC
TGGGAATTTGCAAATGAGGGTTTCTTAAGGGGTCAAAAACACCTTCTTAGAAGTATTACTCGGCGCAAACCTGCCCATGGGCA
TGGTCACCAACAATC
ACAGCAATCACATGGACAGAGTTCATCTGTTGGTGCCTGTGTTGAGGTTGGGAAATTCGGTCTGGAGGAAGAGGTCGAGAGAC
TTAAGAGGGACAAGA
ATGTGCTTATGCAGGAACTAGTTAGGTTGAGGCAGCAGCAACAGGCTACAGATAACCAGCTGCAAGCCATGGTGCAGCGTCTT
CAGGGTATGGAGCAG
CGGCAACAACAGATGATGTCATTCCTTGCAAAGGCTGTGCAAAGCCCTGGTTTCTTGGCGCAGTTTGTACAGCAGCAAAATGA
CAGTAACAGGCGCAT
TACAGAAGCAAACAAAAAAAGGAGGCTCAAGCAGGAAAATATTACTGAGAATGAAGGTTCTGCTGCTCCTGATGGACAGATTG
TTAAATATCAGCCTT
CAATGAATGAGGCAGCAAAAGCAATGATCAGGCAGATCATGAAAATGGAAGCTTCTTCCCGGTTGGAACCCTTCAGTGATGAT
CATGATAATCTCCTG
ATTGGTGATCGCTCTTCGTCATCTACACCAATGGAAAGTGGGAGTTCTTCAAGCCGTATGTCAGGAGTAACCCTTCAAGAGGT
TCCACTGACTTCAGG
GCAGTCATCCTATATGCATGCAACTTCATCTATGCCTGGCCCATCAGCTGCCATCGCTGAAATGCCATCCTCCTTGCCTGCTG
CTGCTTCTGAACAGG
TTAAAACAACTCAATTTCCAGATGTGGGTGTACTGGTTGGGTCACAAGAAGCACCCTCCATCTCTGCTCCTTCCTCAGATGTA
ATCATGCCACAGCTT
TCTCAAATACCAGGAATGGTGCCTGAAAGTATGGTTGATATCCCTGTAGAAAATTTCGTGGGACCTGAGACAGGGTCTGGTGC
ATTTATAGATCCCAC
ATCATTGGTTAATGGTTCAGTGCCCATTGAAATTGATGACATTTCTCCTGATCCTGACATTGATGCTTTGCTAGATAATTCTA
CTTTCTGGGATGAGC
TCCTTGTGCAAAGCCCGGTGCCGGAGGATATGGAATCAACTTCAGTGGACTGTGAAAACAAGGGAAATGAGATGCAACCCATG
GAGAACGGATGGGGC
AAAGCTCATATGGATAAGCTCACAGAACAGATGGGTCTTCTTACGTCAGACACCAAGGGGATCTAG
>MolHSF13
ATGGATGGAGTCAGTGGCGGCAGCAACTCTTGTAACAGCGGTGGCGATGCGTCGACCACCGCTGGAGGCGCAGGGGCGC
CGCCTCAGCCCCAGCCGGTGGCGATGCAGAGCGTGAACTCGCCGCCGCCGTTCCTGAGCAAGACATACGACATGGTGGATGAC
CCCTCGACAGACGGG
ATCGTGTCTTGGAGTCCGACCAACAACAGCTTTGTCGTCTGGAAACCTCCAGAATTCGCGCGGGATCTGCTTCCCAAGTACTT
CAAGCACAACAATTT
CTCAAGCTTCGTCCGCCAGCTTAATACCTATATGTGTTTTCAGCCTGGAGTGAGGTTCACTAGATTCGAAGCTGTTAGATGGA

CAAAGAAAGTTTGTG
ATAGAGTTTGGAGTGCAGTCTTGGGATTAGTGAATGATACTACCTTAGAGCTAGTTGAGTGA
>MolHSF4
ATGAATCCAGAAGACGAGCCAGGTTCAAAATCCCCTCCTAAATCCTCCATCGATGCTACTACTGCTAATACAGCTTCTG
TTCAATCCGAAACAACGTCGTGCTCATCCCAACCTTTGTTCGTGAGTACTCAACAATTTCCCTCTGGGTCTCTCTTCGTTGAC
ACAAGCATGGCTTTT
TGTGGGTCTCCATCTTCTTCTCCATTCTCTTCTCCACTGACAGAATTCGAGGGGTTTTCTACAGTAAACCCACCGCAGTCCTC
TGCTTCTTTATCGCG
GATGTATGAATTCGAGGCAAGCCCAGTTGGTGAAAGCACTCCAAACTTCCCATCGTCTACTGGCGGTGAAACTGAGAGCTTTG
ATGTGCCCCAGCCCC
TGGAGTGCTTGCAGGGCAACCCTGTACCGCCGTTTCTGTCCAAGACTTTCGACCTCGTGAATGATCCGACTCTGGATCCGATA
ATATCCTGGGGCCCA
ACTGGAGAGAGCTTCGTTGTTTGGGAACCAGTGGAGTTCGCGAGGCTCATTCTTCCTCGGAATTTCAAGCATAACAACTTTTC
CAGTTTTGTTCGGCA
GCTTAATACTTATGTGGGTATTGCGATAACACAGCCTGTATGGGCTGGTGTGGTCTGGCTTTCTTCTTGTGAATCTCATGTCT
GTCAGGGTTTTCGCA
AGGTTAATACAGATAGGTGGGAATTTGCAAATGAAGCTTTCCAGCGAGGGAGGAGGCATCTTCTGAAGAACATTCAAAGGCGC
AAGACACCCCAATCC
CAACAGGTAGGCAGCAATATTGGGGCTACTACTGAGACAGGGAGGTCTGGATTGGAAAGCGAGGTAGAAAGATTGAGGACGGA
GAGGAGTTCCATGAT
GCAGGAGGTACTAGAACTACTGCGGCAGCATAAAGGAACAGCTCGTCATATGGAAGAAGTGAACCAGAGGCTGCAGGAAGCAG
AGCAAAGACAGAGAC
AAATGGTTTCGTTTCTGGCAAAGATGTTTCAGAATCCAGCTTTTTTGGCCCGTCTTAAGCAGAAGAAGGAACAGGGAGAAATT
GGCTCTTCAAGGATG
AGGAGGAAGTTTGTCAAGCATCAGCAATTTGAACCTGGTAAATCAGATTTGTCCATGGAAGGGCAGATTGTGAAGTACAAACC
TGAGTGGAGCAACCT
CATCATGTCTTCTTTAGCTCCAGATTCACCTCCAGTTGGTCTGGAGCAGGTTCCTGATTATGCCATGCATGAAGGCATGCCAT
TTCAAGTTGAGAATA
TGCTATCAGAGGAACTAGCCACATTGGAGGCATCAGCACCACCTCAAGTGTTCATGAAAACACCAGAACATTTTGGAGAAGGC
ACATCAAGCTTGGGA
AGTGAAGATCCACTCTTCAGAGGAAAGAAAATAATGAGCCCAAGGCATGAGGTTAGTCCCGAGTACTTTGTCACTCTCCCTGA
GGACCCAATGATGGA
GAAGAATATTTCAGACTTCCCATCTGCTGGAATGGAAGCCTTTTTGAGAGAAGATGATATATGGAACATGGGTTTCGATGCCA
GTGCCATTTTGCCAA
GTTCTAGTGATGAGTTATGGTGTAATCTTGGCAACTACGATGTGCCAGCAGGGGGAATTGCTAGTGTATTATCTGATGTTTGG
GATTTGGGATCTGAA
CAAGCTCCAGGAGGTTCAGGTTTCCCTGATGATTCCACCCTCCAACAGTCTAAGGATCAGGCTGACCAGAAAAAAAGATGA
>MolHSF5
ATGTCAGAGTACGTAAGAAAATCAAGCCCACCACCATTTTTGCTGAAAACTTACATACTGGTGGAAGATCCGGCCACGG
ACGAGGTTATATCGTGGAACGGAGAGGGGACGGGGTTCGTGGTGTGGCAGCCTGCCGAGTTTGCTAGAGACCTCCTCCCTACG
CTCTTCAAGCACAGC
AACTTCTCTAGCTTTGTGCGCCAACTCAACACCTATGGATTTCGAAAAGTTGCAACGAGGAGATGGGAGTTTTGCAATGACAT
GTTTCGAAAGGGAGA
GAGGGAGCTATTGTGCAACATTCGTCGCAGAAAAGCGTGGCCTAAACATCAAGCTGTTAATATTGCAGCACCAATCACATTCC
ACGCCATACCACAGG
AACTTGATGAAGATCAAAGATCCTCATCAACTTCTTCATCTTCAGGTTATGGTACTCTCATGGATGAGAACAAACGTCTCAAG
AGGGAAAATGGGGCT
CTGAGCTCTGAACTTATGACCATGAAGAGGAAGTGCAAGGAGCTTCTTGATTTGGCAGCTAACTATGCAGGGCCAAATGAGAA
ACAAGAGCAAGCTGA
TCATCAGAGACTGATGTTGTTTGGAGTAAGGCTGGAAGTTGAAGGGGATGATCAGTTGAGAGAGAGCGAGAGAAAAGGAGCTG
AGATCAGTGGAAACA
CAAGAAGCTTTTTGCTATCTCAATCATGCCAGTAA
>MolHSF10
ATGCCACCTATCATAGGAGATATACAGGGATATGATAGAAATAACTTCACAGAATTGAGGTTGATATCTGAGGACATCA
AAAGCCTTAGCTTGACAAGCTTGTGGAGGGAATTAGTAACTCTTAATTTTCTTCAGGGTTTTAGAAAAATTGATACAGATCGT
TGGGAATTTGCAAAT
GATGGATTTGTCAGAGGTCAAAAGCATTTGTTGAAGAATATCCCTAGAAGGAAAAATTCTCAGGGTGTTGACCAGCGAAAACC
AGCGCATGCAGTGGA
CAAGTCTGTTGAACCATGTGAAGAAATTGAAAAGACTGAGCTATGGAAGGAAGTTGAGAACTTGAAGACTGATAAAATGGCAC

TTACGCAGGAATTAA
TTAACCTTAGGCAGCACCAAGAGACTACAGATAATAAGTTAGTGCTTTTGAAAGATCGTGTTCAAGGAATGGAGAAAAATCAG
CAGCAGATGCTGTCA
TTCTTAGTGATGGCTATGCAAAGCCCAGGCTTTTTAGTTAAGCTGCTTAATCCCAAAGAAAATAACTGGCGGATGGCTGAGGC
AGGTGCTATGTTAGA
ACAAGTTGCAGAGGAGGCACTGCCTTCTGACAGTATGATTGTAAGGTATCAGCTCCCACTGGATGAAGGACGTAAGCCTGTTC
TCACACCAGTGACAG
ATTCGGAAAATTTCCCTGAATCTGATAATCTTTCTGATGGGCTGAAAGATTTTCTAATGAATGCTGACATCATGAAGGTCCTC
ATGGATGAAAATCAT
GCTCCATTCATTCTGCCAGATTTATATGATGATGGTTCATGGGAAAAGCTCCTCTTAGCTAGTCCCTTCAAAGAAAGCAGTGA
GGACGCTAAGCTGGA
TAAAGAAGGGTCCACAGACTCTGAGGTGGAAATGGAATCCATAGAGTCTGGAAGCCAGTCTGAGAAAACCCCTAATTTTGAAC
TTTGGCTACAACAAA
TTGGGGAATCTCATGACTTTGAAATTGAACCAAGCATAAATGGATCTCACCCGGTGAACTATGGAAATCTGGAACTTCTAACT
GAGCAGATGGGACTT
TTGGCTTCTGATGAAAGTAATTACAAAATATAG
>MolHSF11
ATGGTGAAATCGACAGATAATGGGTCTTCGTCAGTAGCGCCATTTTTGAAGAAATGCTATGAGATGGTAGATGATGAAG
CTACAGATTCGATAATTTCATGGAGTCAAAACAGTGGCAACAGCTTCGTTATTTGGGACATGACTGAGTTCTCTGTGCAGTTG
CTGCCTAAGTACTTC
AAGCACAGCAATTTCTCCAGCTTTATCAGGCAGCTCAACATCTATGTGAGTCTCTTTCTTCTCCTCTATATTCGCTTGATGGG
TGGTTTCTTTTGCTT
GAATTTATGTTCTTCCGGCTTGTGTTGGGTTGAATTTGGTGGCCTGATTGCCTTTTGA
>MolHSF6
ATGCCGGGAGAGCAAACCGGCGAGTCACCTGCCGGTGACTCGCAGAGGTCGCTACCGACTCCGTTCCTCACCAAAACCT
ATCAGCTTGTGGACGATCCGTCCGTCGACGACCTGATTTCATGGAACGAAGACGGATCCACGTTCATCGTATGGCGGCCCGCC
GAATTCGCGAGAGAT
TTGCTCCCCAAGTACTTCAAGCACAACAACTTCTCAAGCTTCGTTCGCCAGCTTAACACTTACGGATTTAGAAAGGTGGTGCC
GGATCGGTGGGAATT
CGCCAATGACTGTTTTAGAAGAGGAGAGAAAGGGCTGCTGCGTGATATACAGCGGCGGAAAATCTCGCAGTCAGCGCCTTCTG
GGGCAGTGACGGTGG
CAGCGCTGCCTGCAGCGGTGTTGCATGCCGTCTCTCCTTCGAATTCCGGCGAGGAGCAGGTGATCTCAGCAAATTCGTCTCCG
CTGGTCGCGACAGCA
GCTGTCGGTGCCACGTTGCAGCGCACCACAAGCTGCGCCACCGCGCCGGAGCTGATGGAGGAGAACGAGAGTTTGAGGATGGA
GAACACGCGGCTCAA
TGACGAGTTGACTCGGCTAAAGGGATTGTGCAACAACATACTAGCGTTGATGACTAATTACGCCTCTGGTCAAGTAGAGAGCG
GGAGGTCCCCAGAGG
GAAAGCCCCTGGAGCTATTGCCGGCGAGGCAAGGGCTCCAGCCGATGGAGGAGGATACCGCCGCCGAAGGCGGCATGAGGCCC
GAGGAAGAGGTGTCC
CCGAAGCTCTTCGGAGTCTCGATTGGCATCAAGCGCCAGAGGAGAGACGAGGAGCTAGAGGAGGAGGGnnnnnnnnnnnnnnn
nnnnnnnnnnnnnnn
nGATGGTGAGACTGGGTCGGACGTGAAATCAGAACCATTGGATGGGAACTCTGATAATCAGGAATCCACGTGGCTGGAACTGG
GCAAGTGA
>MolHSF16
ATGGGGACGGAGGCAAGCAACGACGCCGCACCGTTCGTTGTGAAGACTTACCTGATGGTAAACGATCCGACTACGGATA
TGTTGATCACCTGGGGCAGAGCCAATAACAGCTTCATCGTCGTCGACCCTTTAGATTTTTCGCAGAGGATCCTTCCTGCTTAC
TTCAAGCACAATAAC
TTCTCTAGCTTTGTTCGCCAACTTAACACCTACGGTTTCAAGAAGGTTGATCCAGATAGATGGGAGTTCGCGAACGAGTATTT
CTTACGAGGGCAGAA
ACATTTACTGAAGAACATAGTGAGAAGAAAGCACGCCAAGAGCACGTACGTGCAAGTGATGCAGACAGAAGACTTTTGGGACG
ATGAAGAGATAGTCA
TGGAGATAGCGAGGTTGAAGGAAGAGCAGAAGGCCCTGGAGCAGGAGCTGGAAGGCATGAACAGGCGTTTGGCGGCCACAGAG
AGACGCCCGCATCAA
ATGATGACTTTCCTTTACAAAGTGGTGGAGGATCCCGACCTCCTCTCTCGCATGATGCTCCGGAAAGAACGCACCAAACAGCT
TACCACCGAGAAAAA
ACGACGCTTGATGATATCCTCCCCCAAGTCTGATCACGAAGAAGAAGAATATGCAGGAGCCCTACGGGTAACGTCATCGTCGT
CGCCGGAAAGTGGGT
TGGAAGCGGAGAACATTTCCCAGTCATCACTGTCGCCCGAGAACTCCATGTGGTGGGCAGGACCGACTCATAGACCACAGGAA
ACTAACTACAGTATC

CTACCGGCAGTTATAAGCACCGGTGTGGTGGTGAACGCTTCAACGGCGCCGTGTTGGCCGTCGACCTCGCTGTTTCCGGGGAT
TAATAACGGCATACG
TGGAAGTTATGATGTGAATGGACAGAATATAAGTTATTCGGAGGAGATGACGGCTGCGGTGGAGTCCAGTCCACCACTGCCTT
ATCCGTTTTCGCTGT
TAGGCAGCGGCTTTTAG
>MolHSF17
ATGCAAGAGAGGGACCGACAACACCAGCTCCACAGCAGCCTCATCATTTCCATCGTCAAACTCGCAGGGGAAGATCCTA
CGCTTCTCTGTCGTTTTCCTCTGCAAGATAGATATATCTCGAATGCCGGTAAGTACGGGCTCTCTGGATATTTGTCCACTTCG
ATAACTTTAACAGGA
CAAGTGAGGAGCCAGACCCGGACAGCTAAAGACAATTGTACCCCGGCTGCTAGTACTGCCAATACTGGTATATACCGAAAGAC
ATTCTGTCCGCCGAT
GGGTGATGACGTGGCTCACATCTATTGGCTAATGACTGTAGTCCATAGTGCCGGAGACACCTGGAATTTCAGCAAGCATACAG
CATCCGAGTTTCTAC
TGATCCAAGGCGGCAGAGAGACGTGGCGACCCCTCCCCTTGCCCTTAAGTGGACACGCACCGTGTCACACCATCCCATTTGCC
TCCATTGTCCTACTC
GAATTTCTGTTTCACAAAAAAGAAAACAACAAAACCTCTCTTTCTCAAATCACGCTCATAGCCAGCACCGTTGTCACTGTAGC
TGAGAATCGAGTCAG
TTGGAGAAAGGAAAAGTTGTTTTCATCGTCTCATGATTTACCACGATCCATCGAAGAACCAAGAAACGCCTACACAGAAGTAG
AGAATGTCAAGGCAA
AACCCATTGATAAGGTTGCGATCAAACGAGAGCAAACGACAGCAACACCGCCTACAGAACCAGACAACGATTGCACTGGAAAT
GACGGCGATGATGGC
GGCGGCCGCTGCTGTTCGTCGTCTCCGTCTTCACCCTTGATGAAGAGCTTGGCAACGGCAAGGGATGAGGCTGTCTTGCAAAT
CAAAGAAGAGAAGGA
GATAGAGGTCCTTGATTGTGATCATGTGAACGGTGGAGGTGATGGTGGTGATTGCCTTGGGGTTGATGATAATGGTGATAACG
TCAATAATGGGAACG
GTTCGTGCTCCTCGTCTTCATCAGATGTTTTGCCCAAGCCAATGGAAGGGTTAAACGATTTGGGTCCACCGCCGTTTTTGAAG
AAGACATTTGAAATG
GTGGAGGATCCTGAGACCGACCCTATTGTGTCATGGAGCTTGACTCGCGACAGCTTTATTGTTTGGGATTCGCACAAGTTCTC
TGAGAGGCTGCTCCC
TAAATTCTTCAAGCACAAGAATTTCTCTAGCTTTATTCGCCAACTCAACACTTATGGCTTCAGGAAGATAGATTCAGATAGAT
GGGAGTTTGCTAATA
AAGGCTTTCAAGGGGGGAAGAAACATTTGCTGAAAAACATCAAGAGAAGAAGTAGATACAACAAGCAGCTGGAGAGTGCAATG
TCCGGTTCCAATTCT
GTAACAGTTGGATTGGAAGCAGAAGTTGAGACTATGAAGAATGACCAAACGACACTGAAAATGGAAATCATGAAACTAAGGCA
GCAACAGGAGGAGTC
ACAGCATCAACTCAGAGCTGTTGAAGAGCGCATGCTCTTTGCAGAGTGTAAGCAGCACCATATGCTCAATTTCTTTGCCAGGG
TAGCCAAGGACCCCA
AATTTGTCCAACGATTAATCCAGAAGAGAAAGCTACAAAGAGAGCTTGATGGGGTTGAATATGGCAAGAAGCCTAGGCTACTT
GACACCCAAGCCAGC
AAGAGCTTGCCAGAGGCCGTTGATGCCAGGCAATATGCCAATTGCAGGAACTATACTTTGGAACAATTGGACACGACACAATC
TGAGCTTGTTGGGGT
TTTGTCAGAAAGTATGATTACCAGCCCAGGGCAGGAGCTGCTCTCAGTTCCTACAGGTGATGAATCAGGAAGCCGTACTCAGG
ATGATGGAGAGGATG
TCATGTGCGGAAGGAGTACCTCAGTTACACCTTCTGTTTATCATGAAATATTTGAGAACTTAATGGAGGAAGCTGCAATTAGA
GAAAATGTGGTTAAT
GAAGAAATGACAGTGAATGATTGTAAAATCTATCTTGAGTTGGAAGACTTGTTGGAAAAGCCACAGAATTGGCTGGGATGTAT
AGGTGGGCTGGTTTG
A
>MolHSF18
ATGGTCTCACACGGAGGGGGAAGGGGAGGTAGCATCTGCCTTGCCTGTATCCAGACTGAAAATTTGGCGCATTTTGCTG
CAAACTCGAGTGTTAGTCATCAAGAAGTGTCAAGGAAGCGAAAGGGTATGGAGGGAGTTCCAGTGAAGGAAGAAGACACTGTA
ACTTGTACTGTTAGT
GGTGGTGCATCGTCATCTTCATCATCTTCAAGCTTCTCTCCGCAGCCAAAAGAGGGGCTGCACGAGGTGGGTCCGCCTCCCTT
CCTCACAAAGACCTT
TGAGATGGTGGAGGACCCATCGACGGACTCGGTGGTTTCCTGGAGCAGCAGTCGAAACAGCTTCGTTGTTTGGGACTGCCACA
AGTTCTCTACAACTC
TGCTCCCTAGGTACTTCAAGCACAGTAATTTCTCCAGCTTCATTCGCCAGTTAAACACATATGGTTTTAGAAAGGTCGATCCA
GATAGGTGGGAGTTT
GCCAATGAAGGTTTTCTGGGAGGGCAGAGGCATCTGTTAAAGACCATTAAGAGGAGAAGACATGTAACGCATTGTACCCAACA

ACAAGGAGGAGGCGC
ATGTGTTGAGCTGGGGCAATATGGCTTTGAAAGGGAGCTTGAGAGACTAGAAAGGGATCGAAATATGTTAATGACAGAAATCG
TGAAACTGAGGCAGC
AGCAACAGAATTCAAGGCACCAAATTGTTGCAATGGAGGATAGGTTACGAAGCACTGAGAGGAAACAGCAGCAGATGATGACA
TTCCTTGCTAAGGCT
CTCAATCAGCCTTCTTTTATCCAGCAATTTTTACAGAGCGGTGCACAGAGAAGACAGTTCCAAAGTGTTGAGATTGGGCGAAA
ACGGAGACTAGCTGC
TAGCCCAAGTGTGGAGAATTTACAAGAAGCTGTCCCTGTGGCAGTCGGTAGCAGCCAAGTGGTGGATTATGCAAACCAAGATC
AAGAAGACTTGGCCA
CTATTGAGACTGAGATTGAGACATTATTCTCTGCTGCCATGGACAATGAATCAAGCAATGATATCAAGGACCCTGGAGCAGGT
ACAATACCCACAAGT
GGTGGCAATTTTGATTCAGCCAGCGAGACAATCTGGGAGGACTTTATCAATGAAGATGTAATTGCTGGGAACCCAGAGGAACC
CGCTACAGTGCTGGT
CGGTGAGGAATCAGAAGTTGATGTGGAAGTGGAAGATTTGGTAGCGAACTCCCCAGATTGTTGGTCTCTGCAACTCCATTCCC
ATTCCTCTACATGGG
AATGGAGAAAGTCGCGAAGGGGCCAACAGAATTTCTCTTTCTTCTGCAGGCCTGTAATCGCGGACAACGAAGGAATGGTGTGA
>MolHSF7
ATGATGGCTCCGCCGCCGGTGGAGCAGAACGGAGACTCGATGACGGGAGAACCCCAGAGGACTCTACCGACGCCATTTT
TGACGAAGACGTATCAACTCGTCGACGACCACAACATCGACGATGTTATTTCGTGGAACGAGGATGGATCCAGCTTCATCGTG
TGGAACCCTACGGTA
TTCGCTAGAGATTTGCTCCCCAAATGTTTCAAGCACAACAACTTCTCTAGCTTCGTTCGTCAACTCAACACCTACGGATTTAG
AAAAGTGGTGCCTGA
TCGATGGGAATTCTCGAACGACTGTTTTCGCAGAGGAGAAAAGCAGCTTTTGTGCGAGATTCAGCGACGGAAAATCTCAACGT
CTACGCCGTCCCAGG
CGACGGCGACCGCGTCGGGCATCCCAGTGGCGAAGCCGATGATATCGCCGTCGAATTCGGGAGAAGAGCAGGTTATTTCGTCT
AACTCTTCGCCACCA
AGACCAGCCCAAACAGGAGGCGCTGTTGCATCTGAGCTATTAGAGGAGAACGAAAAATTGAGGAAAGAGAATGTACAACTCAA
TCAAGAGTTGACACA
AATGAAGAATCTCTGCAATAACATCTTTTCTTTGATGTCAAATTACGTTACTTCTCAACCGGAGGCCGGTTTTTCCGCTAGTA
ACACCGGATCTGAAA
GCGGCCTCGAGGCAGTAAAACCGCTGGATTTGCTGCCGGCCAAACGTTCTTCTTGCCAGGGCGAGGCGGGAGAGGAGGACGTG
AGCCCCATGCTGTTT
GGCGTGCCGATTGGCGTGAAGCGCGCAAAGGATGGCTTGCATGTGGAGGGGGAGGAGCAGATGGAGATGCAACTGCAGTTGCA
ACAACCTGGGGGGGA
GATAAAATCAGAGCCATTGGATTGCCAGGGCGGCGATGATGATGGGCACACTCCATGGCTAAGGCAGCGCCATCGAGTCAATC
AGAGGGTGTGCAATT
GA
>MolHSF8
ATGGCGCAGAGATCAGTTCCAGCGCCGTTCCTGACGAAGACGTACCAGCTAGTGGATGATCCAAGCACCGACGACGTCA
TATCGTGGAATGAGAGCGGCACCACTTTTGTTGTATGGAAAACTGCCGATTTCGCCAGGGATTTGCTCCCTAATTATTTCAAG
CACAACAACTTCTCC
AGCTTCATTCGCCAACTCAACACTTACGGCTTTCGGAAGATCGTGCCGGACAAATGGGAATTCGCCAACGAGTACTTTAGGCG
AGGGCAGAAAGAGCT
GCTGGCGGAGATACGACGCCGTAAGACTGTCACGCCGTCGCCGCCAAATGCTCCGGCGAGTGGGAAGTCCGGCGGTGCGGCTC
CCGCGTCGCAGTCGA
ATTCTGGGGAGGACCTGGGGTCAACCTCCACGTCGTCTCCGGACTCAAAGAACCCGGGGTCCTTGGAGACGGCGACCACTGCC
CAGTTCGCGGATTTA
TCGGGGGAGAACGAGAAATTGAGGCGGGACAATGAGATCCTGAGCTCGGAGCTGGCGCAGGCGAAAAAGCAGTGCGACGAGTT
GATCGCGTTCTTGAC
GGATTACGTGAAGGTGGGGCCCGAGCAGATCGATCGCATCGTGCGGCAAGGAAGCTGTGGTGCCATCCGTGATGGACCGATCG
GCnnnnnnnnnnnnn
nnnnnnnnnnnnnnnnnnnCGAAAACAACGGACAAGACTCACAAGAGGACTGTGGTGGCGGTTTTAAACTGTTCGGTGCTTGG
GTGAAAGAAAAGAAC
AAAAAGAGGGCCCGGGATGATAACACTGGGATCGGCGGGGGCCGTGCCAAAGAGATGATGACACTGGAATTTCACGCGCCGCT
GCTGAAGAGTAGCAA
GGTCTGTAACTGA

Protein sequences
>MolHSF2

MNPYGRLDEEYPGASSPYSGMPSIATPQPIEGLYDAGPPPFLRKTYDIVDDSSTDHIVSWSRGNNSFVVWDPQIFSRSLLPKY
FKHNNFSSFVRQLNTYV
RKWTRAVIRPCSALASALQCCTISVVKAFMNINFDVFTSNPLLLLNGFRKVDPDRWEFANEGFLRGQRHLLKNIRRRKRTDMQ
ASQQALDPCVEVGRFGL
DGELDRLRRDKHVLMLELVKLRQQQQNTKAHLQVMEEKLKSTEIKQKQMMSFLARAMQNPNFVQQLVQHKDKRRELEEAISKK
RRRPIDQGRSNIVVGEL
DQGGSSGVFVKIEPKEYGDISEFEVSELDKLAVNMQGLSESPSNLEQQHTEKREEHGSKCKDIDEEFWEDLLDEDIQEEMGVL
DVEGEAEEDVNVLVEQL
GFLGSSPQYRN
>MolHSF3
MTVDGLIRESYGIGKPSRSRWRNKPPRKCSGKQLRIFILLLVVVLFFLLVSDRSLSPLPGKIGFSFAVGNYVQDRPRLVAGNF
SRRQEVKAESDKDLCRG
RYVYVHDLPSRFNQDVLKSCRLLNRPTDKSNMCDNLLNLGFGPRIENSDGVLSDRSWYSTNQFLLEVIFHNRMKNYECLTYDS
SLASAIFVPFYAGLDLR
RYLWRLKTSVRDSSGIDLVRWLAGKPEWRRMGGRDHFLVAGRISRDFRRQSDKNSDWGSKFRLLPESQNMSMLSIESSFSKND
FAIPYPTYFHPSSESEI
FRWQDRMRRNRREFLFSFAGAPRSRRKGSIREEVISQCKASTKTCKFLDCDAATQGCEDPVELMKLFQKSVFCLQPPGDSFTR
RSIFDSILSGCIPVFFH
PGSAYEQYKWHLPRNHTEYSVYIPPKQLKSGKHSIEQTLLGIRRDRVEALREEVVRLIPRITYRDPMSKLETIEDAFDLAIKD
RSFPPELQSLLACVYRP
VARMSSDGLIRESCGFGKPGRSRWRNKPPRKCSSKQLWLFILSFVVLSSFLSVSYHSVSPLPSKIGSTFSMENFVXXXXXXXX
XXXXXFDECLCRGRYVF
IHDLPSRFNQDVLKNCQWLKRPTEESNMCNYILNLGFGPQIQNSNGVLSDRSWYSTNQFLLEVIFHNRMKNYECLTNDSSLAS
AIFVPFYAGLDLYRYLW
RPNLSVRDSSGIDLVRWLTGKPEWRRMGGRDHFLVAGRISRDFRRQSDKHSDWGSNFRLLPESQNMSMLSIESSFSTNDFAIP
YPTYFHPSSESEILQWQ
DRMRRNKREFLFSFAGAPRPRTKGSIREEVIDQCKASNKTCKHLDCDAAAHSCEHPVELMKLFQKSVFCLQPPGDSFTRRSIF
DSILAGCIPVFFHPGSA
YQQYKWHLPRNHTEYSVYIPSKQLKSGNHCIEQTLLGIREETVEALREEVLRLIPRITYRDPMSKSENLEDAFDLAIKAFIFN
WGKHMIDGCFLIIKEVE
VYRSIQKPRSRSTTAQVTCREGRMEAVTGTSSSGGGGGGGGPAPFLLKTYDMVDDSSTDEIVSWSSNNRSFVVWNPPEFARLL
LPTYFKHNNFSSFIRQL
NTYEILLGLLELDFVVDACYMRIDFQEVGFRKIDPERWEFANEDFIKDQKHLLKNIHRRKPIHSHTHPHGSMVDPERAAFDEE
IERLSREKAMLEANILR
FKHQRSAEKHQLEDMTRKMDSMEQKQENLLTFLQKAVQNPAFVEHLARKIESMDISAYNKKRRLPRPESSKPVVENSSLDNNS
SSRPEFGNIFHQDFSNK
LRLELSPAISDINLVSHSTECSNEDGGSPQRKISEGDPKDAHTRTEGLSFAPDILELSDTGTCPRKIPLHESSRVHAFQQRLT
STEDVDGHISCHLNLTL
ASSPLPVNKSPYPARMSQHNQVIGKFPDSRFNVNTKEADNRGFLKNRNLADENTTLSSSREAPDNYQGTTAPPGRVNDVFWEQ
FLTERPGSSDNEEASSI
YRANPYEEQEDRRPGQGISRNSRNIAQLTL
>MolHSF19
MNPFYPVKEEYPGSSSSLSADDPTLMPPPQPMEGLHDTGPPPFLTKTFDMVDDPNTNHIVSWNRGGSDFVVWDPYSFSTNLLP
RYFKHSNFSSFVRQLNT
YGFRKIDPDRWEFANEGFLRGQRHLLRNIKRRKAPSQPLPPQQAVGPCVEVGRFGLDGEMDRLRRDKQVLMMELVKLRQQQQD
TRVYLQEMEQRLQFTEK
KQQQMMSFLARAMQNPAFLQQLAQQKEKRLELEEVMTKKRRRPIDQGPGRVGEGESSRSGEGVNSVKSEPLEYGLEVSELEAL
ALEMQGYGRARKEQVEE
QEELELEPGEGGDRELDEGFWEELLSDERFEGELDIPGAEGGEHEDVSVLADRLGYLGSSPK
>MolHSF20
MDNSQGNSSAPPPFLLKTYEMVDDPVTNSMVSWSPSGCSFIVWNPPDFARDLLPKYFKHNNFSSFVRQLNTYGFRKIDPDQWE
FANDEFIRGERHLLKNI
HRRKPIHSHSAQLRDGSSLQLAESEKEEFQKEIEQLKHDKSLLYLELQKRQREIQGFKLQRRSLHDRLQNMEHRQMKLTSFLA
QLLQKPGFASMLLQQSQ
IHTKKRRLLKVDHSNDESNVLEDWKLIVQKENMDESTALILDSQRVEKLDSSLKFWESILHGAGEAFGEVHDSSRLSYPSRVL
VTELHASSEDHDVHVEP
SSPRVPSSSCQLRNVHSSPELAGSMYHVGSPVSHLTADMRPKTLEMDVNPELTSASNAEALDEYTSGTTNPSASTRVNDLFWE
QFLTETPGSFAEQE
>MolHSF9

MALMLENCEGILLSLDSHKSVPAPFLTKTYQLVDDPATDHIVSWGEDDTTFVVWRPPEFARDLLPNYFKHNNFSSFVRQLNTY
GFRKIVPDRWEFANEFF
KKGEKHLLCEIHRRKTAQPQVAINXXXXXXXXHSPLGVNGPGFFPFPSRVSISPSDSDEQANWCDSPPLSSPRGATGVSVVST
ASAAVGGAYNNSVTALT
EDNERLRRSNNMLMSELAHMKKLYNDIIYFVQNHVKPVAPSNSYPSSLLLCNPPPSATTPLGTTNCSLGQKPLNQLLGYYPTN
PKQAPQVQVLNSPTTTA
QSTVTIVEEAKNNSCKTKLFGVPLQSKKRLHPEYGSGQATMETNKARSRLVLEKDDLGLNLMPPSTC
>MolHSF1
MAFTVERCEEMVFSVESQKTVPAPFLTKTYQLVDDSLTDHIVSWGEDGTTFVVWRPPEFARDLLPNYFKHNNFSSFVRQLNTY
GFKKVVADRWEFGNECF
RKGAKHLLSEIHRRKTPQHHHQHYHDQQQQPPFLQPEESFAWIDHCPVPSPKVRTDILTALTEDNQRLRRKNYMLLSELAHMK
TLYNDIIYFIQNHVKPV
VPQEPIRANSASVPKLVELGSSVLQDETLLGIQAVNNGSMGKPSGGSTEERSSIGTVKLFGVPLSGRKRLHSEKIDQQELECG
VIP
>MolHSF14
MDDNQGSSNSLPPFLTKTYEMVDDPSTDSIVSWSQSNKSFIVWNPPEFASDLLPRFFKHNNFSSFIRQLNTYGFRKIDPEQWE
FANEEFIRGQPQLLKNI
HRRKPVHSHSLQNLQGQATLTESERQSLKGEIERLRKDKELLLLESLRHEEEQQGLELQMQLLKERLQHMEKRQQTMVSLVAR
VLQKPALALTLSPRLET
NDRKRRLSRIGYLYDEDTQMGTSQIVARENGDNSSAVSSNTELFEQLESSLVFWENVIHDVDETCIQLNANLEVDESTSCAES
PAISCIQLNVDARPKSP
EIDMNSEPAAVVVPEPVAPKEQQMGTAAAPPTGVNDVFWEQFLTENPGSTDTQEVQSERKDPDGRSDSKPGDGKFCWNLRNVN
NLAEQMEHLTPAERT
>MolHSF15
MPRKFLKLKKSEMTSSPGTKGPTPFLWKTYDLLEEVDRSCCGGKRIVSWNGDGTGFVVWSPAEFSELVLPKYFKHNNFSSFVR
QLNTYGFKKISMERWEF
KHEKFQRGCKHKLVEITRKKKELSVFPAYLKASLDDAAAATQETHRLALMEENKNLRRQKVELQMQIKEVKALEIRSYYTNRV
NKATLYSKISPLGDRTT
VVHELENWVKNGNKVRVAELQRIIHDLRKRKRFTQALEVSLWMNEKGICTFSPSEHAVQLDLIGRVHGFVSAESYFDNLRDED
KTDKTYGALLNCYVRQR
QTDKSLSHLEKMKKMGLASSPLTYNDIMCLYTNIGQHDKVPGVLAEMKENKVSPDNFSYRICINSYGVRSDLEGMERLLQEME
SQSNIVMDWNTYAVAAN
FYIKAGLKDKAIVLLKKSEERLEKKDGVGYNHLISLYASLGNKAEVLRLWHLEKSACKRYINRDYITMLQSLVKLGEVEEAEI
ILREWESSGNCYDFRVP
NTVIIGCAEKGLLDKSKAMLQDLTKKGMTVTPNSWGVVAAGYMKKGQLEKAFQSMKAALSVHLEFKGWKPNLNVITSILNWLG
DKGSVGDSEAFVALLRT
AIPVNRQMYHTLIKASLRDDREVDGILKRMKAHMIDEDDETKKILGTRQK
>MolHSF21
MDSVHDSISIGNYVEANGSSVPPFLSKTYDMVDDPSTDSVVSWSNSNNSFVVWDVPEFARDLLPKYFKHSNFSSFVRQLNTYV
ECIDTELILSIDLDFSV
PMEYLNDGRLIGYRFEVSYIMIKKILSVWLTGLNSEIDQMEAIPLSIASLARLSEQVLPLMPSIAVALTVASTVYYSSAIHVS
FDTWNLRSGAYTELILE
IINYMHRSALFLSGSIALQCRFWMGFRKVDPDRWEFANEGFLRGLKHLLKSIVRRKPAHVHNHHQPQVQNTSVGACVEVGKFG
LEEEVERLKRDKNVLMQ
ELVRLRQQQQTTEQQLQTVGQKVQVMEQRQQQMMSFLAKAMQSPGFLNQLVQHQNDSNRRIAGGNKKRRLPRQGEENLAGEHP
ASGPNGQIVKFQPSMDE
AAKAMLHQIMKMNASPRLEASVNNPGTFLINDISSSNVLENGSSLNGVSGVTLSEVSPVSGQSSISAKLGFPVSGTSTAITEM
QSSPCMVSESGKVDQIP
EMNMHHCGEDIILANSSQVQENMSGNTVGNTDANFMDSEVNAVSILDEKKAIKTKIVSPEHDIDFFMDGAPKLPGINDLFWEQ
FLAVSQLTVDTGEINSS
SLDSCVTKEYELQLDQKYGWDKM
>MolHSF12
MLRAVSCKEIETPSKMPKKSCICWVKDVLSWALSVRFTAFQDFMCWRLWEGFRKVDPDRWEFANEGFLRGQKHLLRSITRRKP
AHGHGHQQSQQSHGQSS
SVGACVEVGKFGLEEEVERLKRDKNVLMQELVRLRQQQQATDNQLQAMVQRLQGMEQRQQQMMSFLAKAVQSPGFLAQFVQQQ
NDSNRRITEANKKRRLK
QENITENEGSAAPDGQIVKYQPSMNEAAKAMIRQIMKMEASSRLEPFSDDHDNLLIGDRSSSSTPMESGSSSSRMSGVTLQEV
PLTSGQSSYMHATSSMP
GPSAAIAEMPSSLPAAASEQVKTTQFPDVGVLVGSQEAPSISAPSSDVIMPQLSQIPGMVPESMVDIPVENFVGPETGSGAFI

DPTSLVNGSVPIEIDDI
SPDPDIDALLDNSTFWDELLVQSPVPEDMESTSVDCENKGNEMQPMENGWGKAHMDKLTEQMGLLTSDTKGI
>MolHSF13
MDGVSGGSNSCNSGGDASTTAGGAGAPPQPQPVAMQSVNSPPPFLSKTYDMVDDPSTDGIVSWSPTNNSFVVWKPPEFARDLL
PKYFKHNNFSSFVRQLN
TYMCFQPGVRFTRFEAVRWTKKVCDRVWSAVLGLVNDTTLELVE
>MolHSF4
MNPEDEPGSKSPPKSSIDATTANTASVQSETTSCSSQPLFVSTQQFPSGSLFVDTSMAFCGSPSSSPFSSPLTEFEGFSTVNP
PQSSASLSRMYEFEASP
VGESTPNFPSSTGGETESFDVPQPLECLQGNPVPPFLSKTFDLVNDPTLDPIISWGPTGESFVVWEPVEFARLILPRNFKHNN
FSSFVRQLNTYVGIAIT
QPVWAGVVWLSSCESHVCQGFRKVNTDRWEFANEAFQRGRRHLLKNIQRRKTPQSQQVGSNIGATTETGRSGLESEVERLRTE
RSSMMQEVLELLRQHKG
TARHMEEVNQRLQEAEQRQRQMVSFLAKMFQNPAFLARLKQKKEQGEIGSSRMRRKFVKHQQFEPGKSDLSMEGQIVKYKPEW
SNLIMSSLAPDSPPVGL
EQVPDYAMHEGMPFQVENMLSEELATLEASAPPQVFMKTPEHFGEGTSSLGSEDPLFRGKKIMSPRHEVSPEYFVTLPEDPMM
EKNISDFPSAGMEAFLR
EDDIWNMGFDASAILPSSSDELWCNLGNYDVPAGGIASVLSDVWDLGSEQAPGGSGFPDDSTLQQSKDQADQKKR
>MolHSF5
MSEYVRKSSPPPFLLKTYILVEDPATDEVISWNGEGTGFVVWQPAEFARDLLPTLFKHSNFSSFVRQLNTYGFRKVATRRWEF
CNDMFRKGERELLCNIR
RRKAWPKHQAVNIAAPITFHAIPQELDEDQRSSSTSSSSGYGTLMDENKRLKRENGALSSELMTMKRKCKELLDLAANYAGPN
EKQEQADHQRLMLFGVR
LEVEGDDQLRESERKGAEISGNTRSFLLSQSCQ
>MolHSF10
MPPIIGDIQGYDRNNFTELRLISEDIKSLSLTSLWRELVTLNFLQGFRKIDTDRWEFANDGFVRGQKHLLKNIPRRKNSQGVD
QRKPAHAVDKSVEPCEE
IEKTELWKEVENLKTDKMALTQELINLRQHQETTDNKLVLLKDRVQGMEKNQQQMLSFLVMAMQSPGFLVKLLNPKENNWRMA
EAGAMLEQVAEEALPSD
SMIVRYQLPLDEGRKPVLTPVTDSENFPESDNLSDGLKDFLMNADIMKVLMDENHAPFILPDLYDDGSWEKLLLASPFKESSE
DAKLDKEGSTDSEVEME
SIESGSQSEKTPNFELWLQQIGESHDFEIEPSINGSHPVNYGNLELLTEQMGLLASDESNYKI
>MolHSF11
MVKSTDNGSSSVAPFLKKCYEMVDDEATDSIISWSQNSGNSFVIWDMTEFSVQLLPKYFKHSNFSSFIRQLNIYVSLFLLLYI
RLMGGFFCLNLCSSGLC
WVEFGGLIAF
>MolHSF6
MPGEQTGESPAGDSQRSLPTPFLTKTYQLVDDPSVDDLISWNEDGSTFIVWRPAEFARDLLPKYFKHNNFSSFVRQLNTYGFR
KVVPDRWEFANDCFRRG
EKGLLRDIQRRKISQSAPSGAVTVAALPAAVLHAVSPSNSGEEQVISANSSPLVATAAVGATLQRTTSCATAPELMEENESLR
MENTRLNDELTRLKGLC
NNILALMTNYASGQVESGRSPEGKPLELLPARQGLQPMEEDTAAEGGMRPEEEVSPKLFGVSIGIKRQRRDEELEEEXXXXXX
XXXXXDGETGSDVKSEP
LDGNSDNQESTWLELGK
>MolHSF16
MGTEASNDAAPFVVKTYLMVNDPTTDMLITWGRANNSFIVVDPLDFSQRILPAYFKHNNFSSFVRQLNTYGFKKVDPDRWEFA
NEYFLRGQKHLLKNIVR
RKHAKSTYVVQVMQTEDFWDDEEIVMEIARLKEEQKALEQELEGMNRRLAATERRPHQMMTFLYKVVEDPDLLSRMMLRKERTK
QLTTEKKRRLMISSPKS
DHEEEEYAGALRVTSSSSPESGLEAENISQSSLSPENSMWWAGPTHRPQETNYSILPAVISTGVVVNASTAPCWPSTSLFPGI
NNGIRGSYDVNGQNISY
SEEMTAAVESSPPLPYPFSLLGSGF
>MolHSF17
MQERDRQHQLHSSLIISIVKLAGEDPTLLCRFPLQDRYISNAGKYGLSGYLSTSITLTGQVRSQTRTAKDNCTPAASTANTGI
YRKTFCPPMGDDVAHIY
WLMTVVHSAGDTWNFSKHTASEFLLIQGGRETWRPLPLPLSGHAPCHTIPFASIVLLEFLFHKKENNKTSLSQITLIASTVVT
VAENRVSWRKEKLFSSS
HDLPRSIEEPRNAYTEVENVKAKPIDKVAIKREQTTATPPTEPDNDCTGNDGDDGGGRCCSSSPSSPLMKSLATARDEAVLQI
KEEKEIEVLDCDHVNGG

GDGGDCLGVDDNGDNVNNGNGSCSSSSSDVLPKPMEGLNDLGPPPFLKKTFEMVEDPETDPIVSWSLTRDSFIVWDSHKFSER
LLPKFFKHKNFSSFIRQ
LNTYGFRKIDSDRWEFANKGFQGGKKHLLKNIKRRSRYNKQLESAMSGSNSVTVGLEAEVETMKNDQTTLKMEIMKLRQQQEE
SQHQLRAVEERMLFAEC
KQHHMLNFFARVAKDPKFVQRLIQKRKLQRELDGVEYGKKPRLLDTQASKSLPEAVDARQYANCRNYTLEQLDTTQSELVGVL
SESMITSPGQELLSVPT
GDESGSRTQDDGEDVMCGRSTSVTPSVYHEIFENLMEEAAIRENVVNEEMTVNDCKIYLELEDLLEKPQNWLGCIGGLV
>MolHSF18
MVSHGGGRGGSICLACIQTENLAHFAANSSVSHQEVSRKRKGMEGVPVKEEDTVTCTVSGGASSSSSSSSFSPQPKEGLHEVG
PPPFLTKTFEMVEDPST
DSVVSWSSSRNSFVVWDCHKFSTTLLPRYFKHSNFSSFIRQLNTYGFRKVDPDRWEFANEGFLGGQRHLLKTIKRRRHVTHCT
QQQGGGACVELGQYGFE
RELERLERDRNMLMTEIVKLRQQQQNSRHQIVAMEDRLRSTERKQQQMMTFLAKALNQPSFIQQFLQSGAQRRQFQSVEIGRK
RRLAASPSVENLQEAVP
VAVGSSQVVDYANQDQEDLATIETEIETLFSAAMDNESSNDIKDPGAGTIPTSGGNFDSASETIWEDFINEDVIAGNPEEPAT
VLVGEESEVDVEVEDLV
ANSPDCWSLQLHSHSSTWEWRKSRRGQQNFSFFCRPVIADNEGMV
>MolHSF7
MMAPPPVEQNGDSMTGEPQRTLPTPFLTKTYQLVDDHNIDDVISWNEDGSSFIVWNPTVFARDLLPKCFKHNNFSSFVRQLNT
YGFRKVVPDRWEFSNDC
FRRGEKQLLCEIQRRKISTSTPSQATATASGIPVAKPMISPSNSGEEQVISSNSSPPRPAQTGGAVASELLEENEKLRKENVQ
LNQELTQMKNLCNNIFS
LMSNYVTSQPEAGFSASNTGSESGLEAVKPLDLLPAKRSSCQGEAGEEDVSPMLFGVPIGVKRAKDGLHVEGEEQMEMQLQLQ
QPGGEIKSEPLDCQGGD
DDGHTPWLRQRHRVNQRVCN
>MolHSF8
MAQRSVPAPFLTKTYQLVDDPSTDDVISWNESGTTFVVWKTADFARDLLPNYFKHNNFSSFIRQLNTYGFRKIVPDKWEFANE
YFRRGQKELLAEIRRRK
TVTPSPPNAPASGKSGGAAPASQSNSGEDLGSTSTSSPDSKNPGSLETATTAQFADLSGENEKLRRDNEILSSELAQAKKQCD
ELIAFLTDYVKVGPEQI
DRIVRQGSCGAIRDGPIGXXXXXXXXXXXENNGQDSQEDCGGGFKLFGAWVKEKNKKRARDDNTGIGGGRAKEMMTLEFHAPL
LKSSKVCN
