# Supplementary material for: De novo Whole-Genome Assembly of Moringa oleifera Helps Identify Genes Regulating Drought Stress Tolerance
Source: Front Plant Sci. 2021 Dec 14;12:766999. doi: 10.3389/fpls.2021.766999 (PMC8712769; doi:10.3389/fpls.2021.766999)
Supplement: Supplementary Figure 1 — Moringa varieties, viz., Bhagya, ODC3, PKM1, and PKM2, during drought stress treatment. [file Data_Sheet_1.zip › Supplementary Table 10.DOCX]

**Supplementary Table S10. Ka/Ks values for tandemly duplicate MolHSFs**

| Seq_1 | Seq_2 | Ka | Ks | Ka_Ks | Effective Length |
| --- | --- | --- | --- | --- | --- |
| MolHSF17 | MolHSF18 | 0.490532628885555 | 2.40915320159821 | 0.203612052799357 | 1215 |
| MolHSF19 | MolHSF20 | 0.645240958502247 | 3.83639022907097 | 0.168189605325551 | 996 |
