# Supplementary material for: De novo Whole-Genome Assembly of Moringa oleifera Helps Identify Genes Regulating Drought Stress Tolerance
Source: Front Plant Sci. 2021 Dec 14;12:766999. doi: 10.3389/fpls.2021.766999 (PMC8712769; doi:10.3389/fpls.2021.766999)
Supplement: Supplementary Figure 1 — Moringa varieties, viz., Bhagya, ODC3, PKM1, and PKM2, during drought stress treatment. [file Data_Sheet_1.zip › Supplementary Tables 1-4 .DOCX]

**Supplementary Table S1: Metrics of the raw data generated on sequencing platforms for *M.oleifera***

| PacBio Sequel Platform | | | |
| --- | --- | --- | --- |
| Analysis Metric | SMRT1 | SMRT2 | Total |
| Polymerase Read Bases (Gb) | 11.22 | 11.80 | 23.02 |
| Polymerase Reads | 659,294 | 718,220 | 1,377,514 |
| Polymerase Read Length  (mean) | 17,027 | 16,435 | 16,731 |
| Polymerase Read N50 | 26,238 | 26,125 | 26,182 |
| Insert Length (mean) | 13,003 | 12,599 | 12,801 |
|  |  |  |  |
| Illumina Platform | | | |
| Samples | MORINGA-350BP | MORINGA-550BP |  |
| Number of paired-end reads | 345,800,986 | 260,464,422 |  |
| Number of bases (Mb) | 51,870.14 | 39,069.66 |  |
| GC % | 38.24 | 38.485 |  |

**Supplementary Table S2: BUSCO analysis for *M. oleifera* whole genome assembly**

| Complete BUSCOs (C) | | | | | 1,381 (95.9%) | |
| --- | --- | --- | --- | --- | --- | --- |
| Complete and single-copy BUSCOs (S) | | | | | 1,360 (94.4%) | |
| Complete and duplicated BUSCOs (D) | | | | | 21 (1.5%) | |
| Fragmented BUSCOs (F) | | | | | 19 (1.3%) | |
| Missing BUSCOs (M) | | | | | 40 (2.8%) | |
| Total BUSCO groups searched | | | | | 1,440 | |
| **Results of LTR_retriever for LAI** | | | | | | |
| Chr | From | To | Intact | Total | raw_LAI | LAI |
| whole_genome | 1 | 281946330 | 0.0031 | 0.0660 | 4.64 | 10.27 |

**Supplementary Table S3. Detailed genome QC statistics for three assemblies of *M. oleifera***

| **Parameter** | **Present study** | **Chang et al., 2019** | **NCBS/TIFR (PRJNA268707)** |
| --- | --- | --- | --- |
| **Total number of scaffolds** | 915 | 22329 | 57169 |
| **Total size of scaffolds** | 281946330 | 216759177 | 253894500 |
| **Percentage of estimated genome represented** | 89.50677143 | 68.81243714 | 80.601429 |
| **Number of scaffolds larger than 25Kb** | 277563747 | 199790068 | 111903472 |
| **Percentage of estimated genome represented in scaffolds larger than 25Kb** | 88.11547524 | 63.42541841 | 35.524912 |
| **Length of largest scaffold** | 13807473 | 4637711 | 235870 |
| **Length of smallest scaffold** | 1056 | 150 | 200 |
| **Number of sequences larger than 1Kb** | 915 | 4012 | 27991 |
| **Percentage of estimated genome represented in scaffolds larger than 1Kb** | 100 | 17.96766537 | 48.96185 |
| **Number of sequences larger than 10Kb** | 676 | 602 | 6231 |
| **Percentage of estimated genome represented in scaffolds larger than 10Kb** | 73.87978142 | 2.696045501 | 10.899264 |
| **Number of sequences larger than 100Kb** | 168 | 292 | 83 |
| **Percentage of estimated genome represented in scaffolds larger than 100Kb** | 18.36065574 | 1.307716423 | 0.1451836 |
| **Number of sequences larger than 1Mb** | 55 | 52 | 0 |
| **Percentage of estimated genome represented in scaffolds larger than 1Mb** | 6.010928962 | 0.232881007 | 0 |
| **Number of sequences larger than 10Mb** | 4 | 0 | 0 |
| **Percentage of estimated genome represented in scaffolds larger than 10Mb** | 0.43715847 | 0 | 0 |
| **N50 value** | 4719167 | 957246 | 20496 |
| **L50 value** | 17 | 56 | 3107 |
| **NG50 value** | 3712021 | 389376 | 12733 |
| **LG50 value** | 21 | 138 | 4999 |
| **Percentage of A** | 31.09336766 | 31.12083416 | 26.219813 |
| **Percentage of C** | 18.93980461 | 18.21219685 | 13.492048 |
| **Percentage of G** | 18.88175952 | 18.28634826 | 13.450373 |
| **Percentage of T** | 31.08481994 | 30.99009829 | 26.042329 |
| **Number of unambiguous bases (N)** | 700 | 3014085 | 52798471 |
| **Percentage of N** | 0.000248274 | 1.390522441 | 20.795437 |

**Supplementary Table S4.** **Orthogroups identified after comparing different plant species**

| Number of species | 12 |
| --- | --- |
| Number of genes | 378983 |
| Number of genes in orthogroups | 326876 |
| Number of unassigned genes | 52107 |
| Percentage of genes in orthogroups | 86.3 |
| Percentage of unassigned genes | 13.7 |
| Number of orthogroups | 27675 |
| Number of species-specific orthogroups | 8711 |
| Number of genes in species-specific orthogroups | 40027 |
| Percentage of genes in species-specific orthogroups | 10.6 |
| Mean orthogroup size | 11.8 |
| Median orthogroup size | 9 |
| G50 (assigned genes) | 18 |
| G50 (all genes) | 15 |
| O50 (assigned genes) | 5129 |
| O50 (all genes) | 6718 |
| Number of orthogroups with all species present | 6574 |
| Number of single-copy orthogroups | 536 |
