# Supplementary material for: De novo Whole-Genome Assembly of Moringa oleifera Helps Identify Genes Regulating Drought Stress Tolerance
Source: Front Plant Sci. 2021 Dec 14;12:766999. doi: 10.3389/fpls.2021.766999 (PMC8712769; doi:10.3389/fpls.2021.766999)
Supplement: Supplementary Figure 1 — Moringa varieties, viz., Bhagya, ODC3, PKM1, and PKM2, during drought stress treatment. [file Data_Sheet_1.zip › Supplementary Tables 7-8 .DOCX]

| GENE | FORWARD PRIMER | REVERSE PRIMER |
| --- | --- | --- |
| \|    \| HSF2 \| \| --- \| \| \| --- \| --- \| | \|    \| GCGACCAATTGATCAAGGACG \| \| --- \| \| \| --- \| --- \| | \|    \| GCTGCTGCTCCAGGTTACTT \| \| --- \| \| \| --- \| --- \| |
| HSF3 | ACTGGAACATGCCCACGAAA | \|    \| GCAATGGCGAAGATGCCAAA \| \| --- \| \| \| --- \| --- \| |
| HSF19 | AAGTAGGAGTGGGGAGGGAG | GCAAGTGCTTCCAGCTCTGA |
| HSF20 | AGTGCCTTCATCCTCTTGCC | GCACTTGTGAGCTCAGGGTT |
| HSF9 | CGCAGCCACAAGTAGCCATC | CGTACTGACAACTGAGACCCC |
| HSF1 | CCCAGGAGCCAATAAGAGCC | GGACCCCAAAGAGCTTCACA |
| HSF14 | AACAAATGGGGACTGCTGCT | CCAGGTTTACTGTCGCTCCT |
| HSF15 | TGTTGTGGCAGCAGGTTACA | CAGTCCTCAACAAGGCCACA |
| HSF21 | AGTCAACAACCCTGGCACTT | GGATGTGCCACTGACAGGAA |
| HSF12 | ACCCTCCATCTCTGCTCCTT | AATGCACCAGACCCTGTCTC |
| ACTIN | TGGAAAGTGTCAAAGTGGGG | CGATAATAACAACAGTAATGGCA |

**Supplementary Table S7. List of primers used for qRT PCR**

**Supplementary Table S8: Statistics of the genome guided transcriptome assembly of *M.oleifera* var. Bhagya**

| **Assembly Parameter** | **Value** |
| --- | --- |
| Total count of contigs | 1,37,132 |
| N50 value | 1221 bp |
| N50 index value | 32020 |
| Average contig size | 932.09 bp |
| Length of largest contig | 16371 bp |
| Length of smallest contig | 258 bp |
| **Results of quality assessment against BUSCO** | |
| Database: Embryophyta_odb10  C:82.2  [S:33.7%,D:48.5%],F:13.6%,M:4.2%,n:1614  1326 Complete BUSCOs (C)  544 Complete and single-copy BUSCOs (S)  782 Complete and duplicated BUSCOs(D)  220 Fragmented BUSCOs (F)  68 Missing BUSCOs (M)  1614 Total BUSCO groups searched | 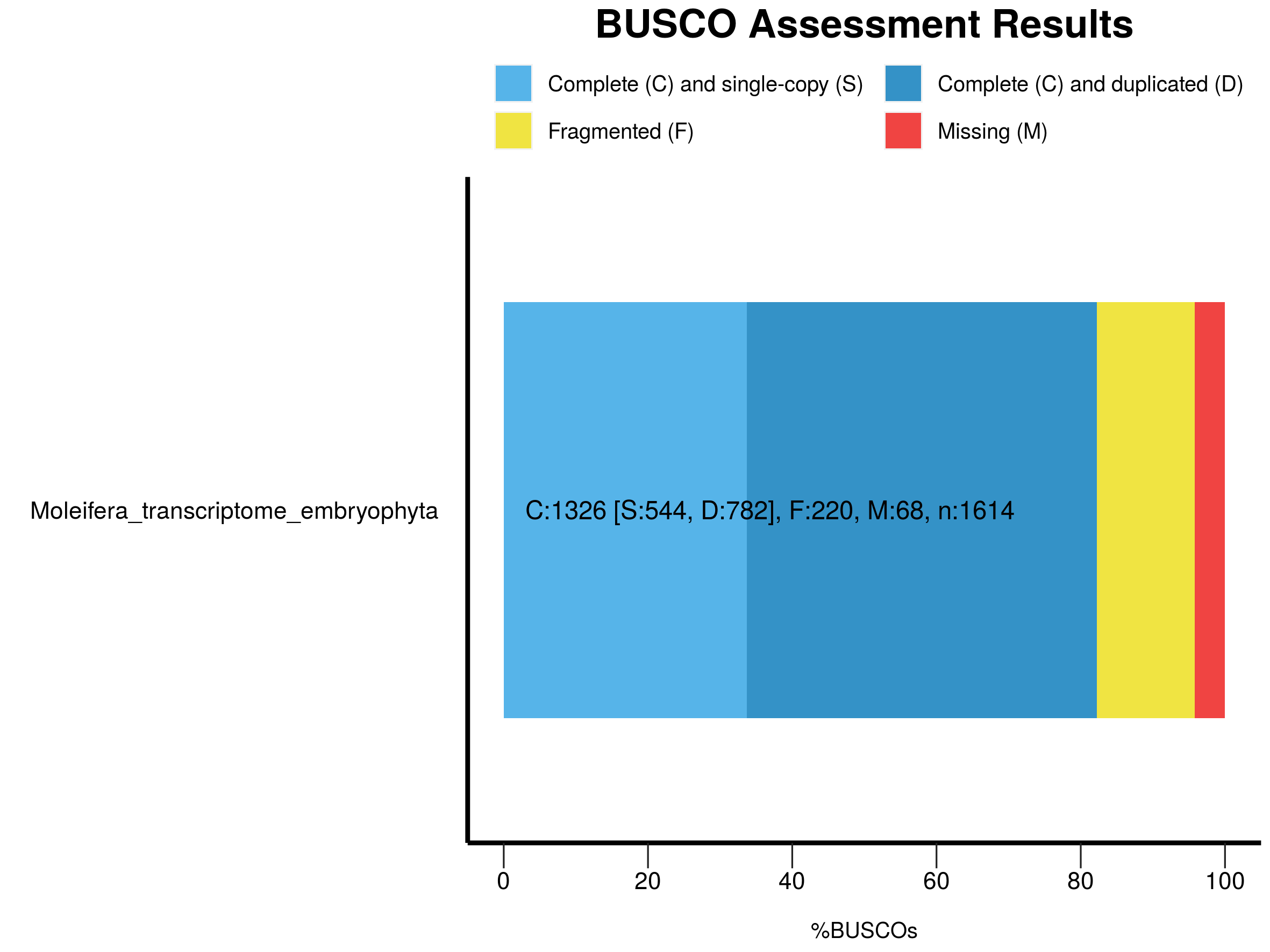 |
| Database: Eukaryota_odb10  C:92.9  [S:34.1%,D:58.8%],F:4.7%,M:2.4%,n:255    237 Complete BUSCOs (C)  87 Complete and single-copy BUSCOs (S)  150 Complete and duplicated BUSCOs (D)  12 Fragmented BUSCOs (F)  6 Missing BUSCOs (M)  255 Total BUSCO groups searched | 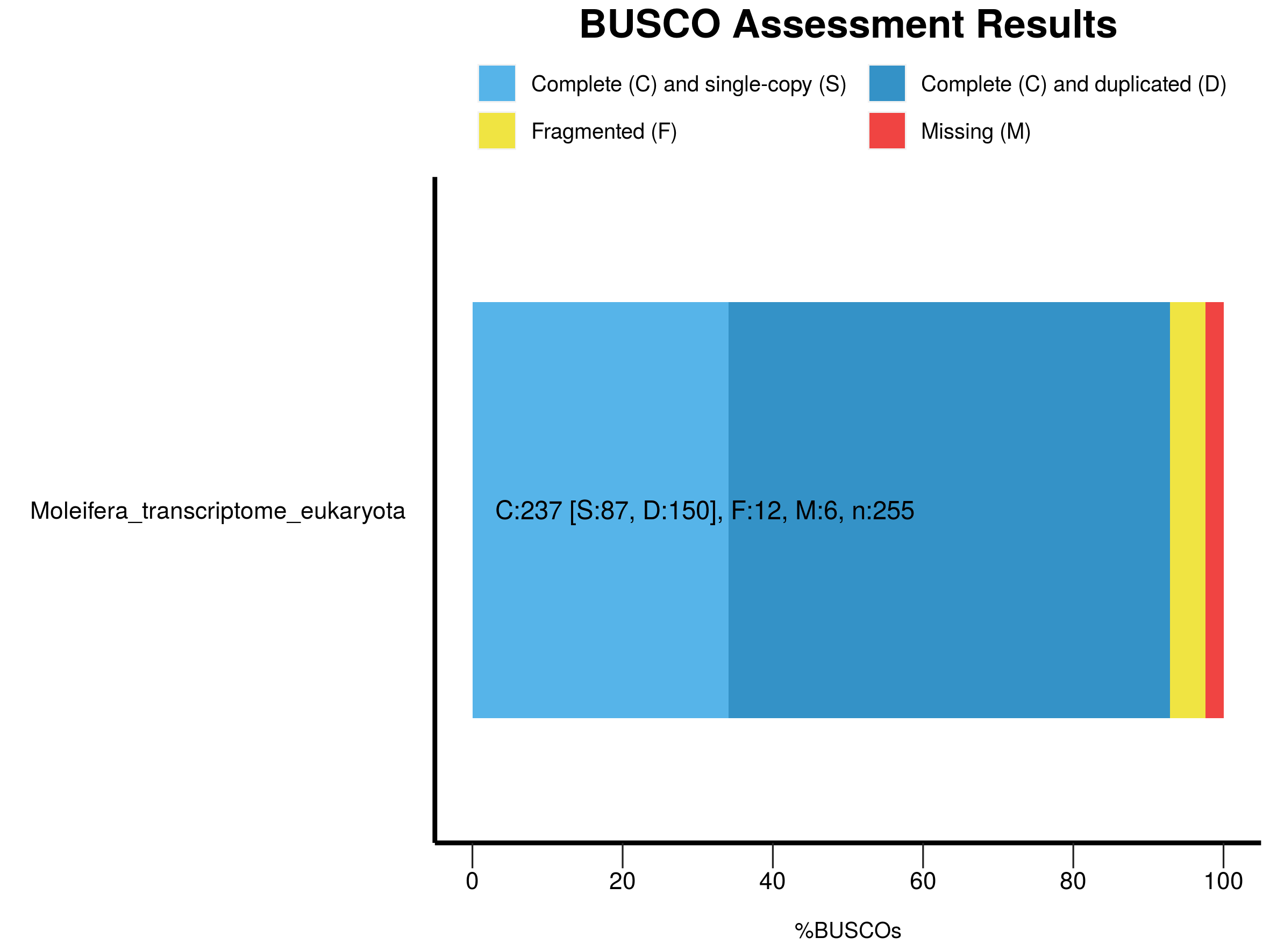 |
