# Supplementary figures and images for: De novo Whole-Genome Assembly of Moringa oleifera Helps Identify Genes Regulating Drought Stress Tolerance
Source: Front Plant Sci. 2021 Dec 14;12:766999. doi: 10.3389/fpls.2021.766999 (PMC8712769; doi:10.3389/fpls.2021.766999)

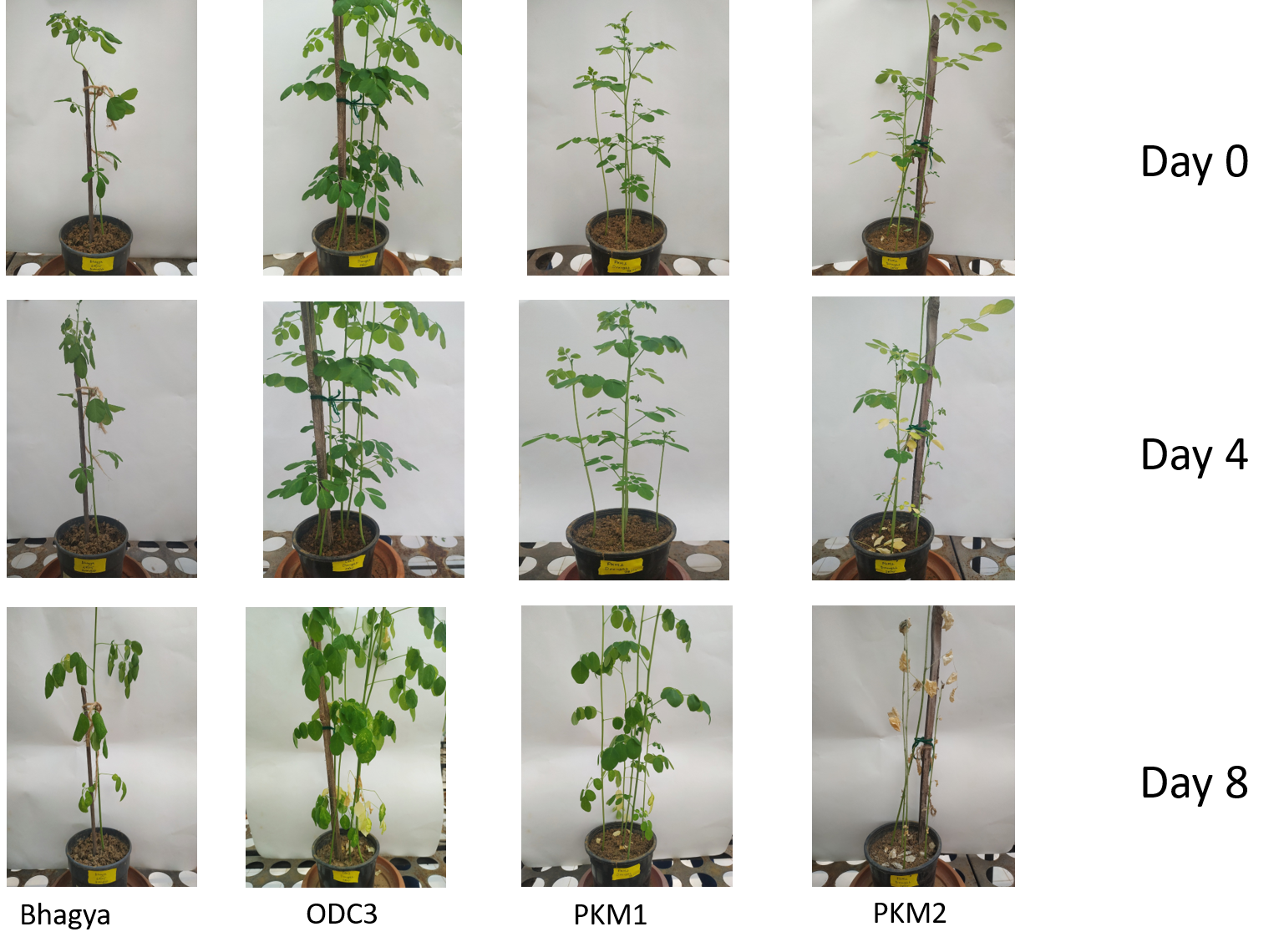

Supplement: Supplementary Figure 1 — Moringa varieties, viz., Bhagya, ODC3, PKM1, and PKM2, during drought stress treatment. [file Data_Sheet_1.zip › Supplementary Figure 1.TIF]

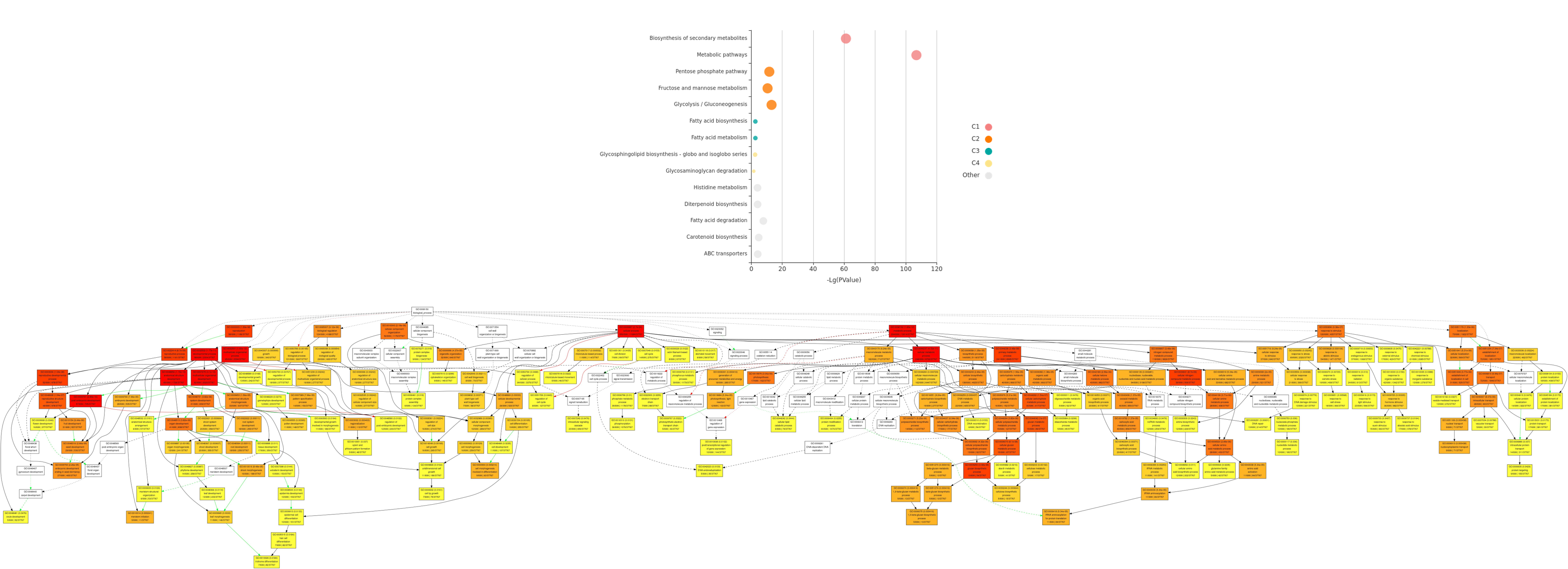

Supplement: Supplementary Figure 1 — Moringa varieties, viz., Bhagya, ODC3, PKM1, and PKM2, during drought stress treatment. [file Data_Sheet_1.zip › Supplementary Figure 2.PNG]

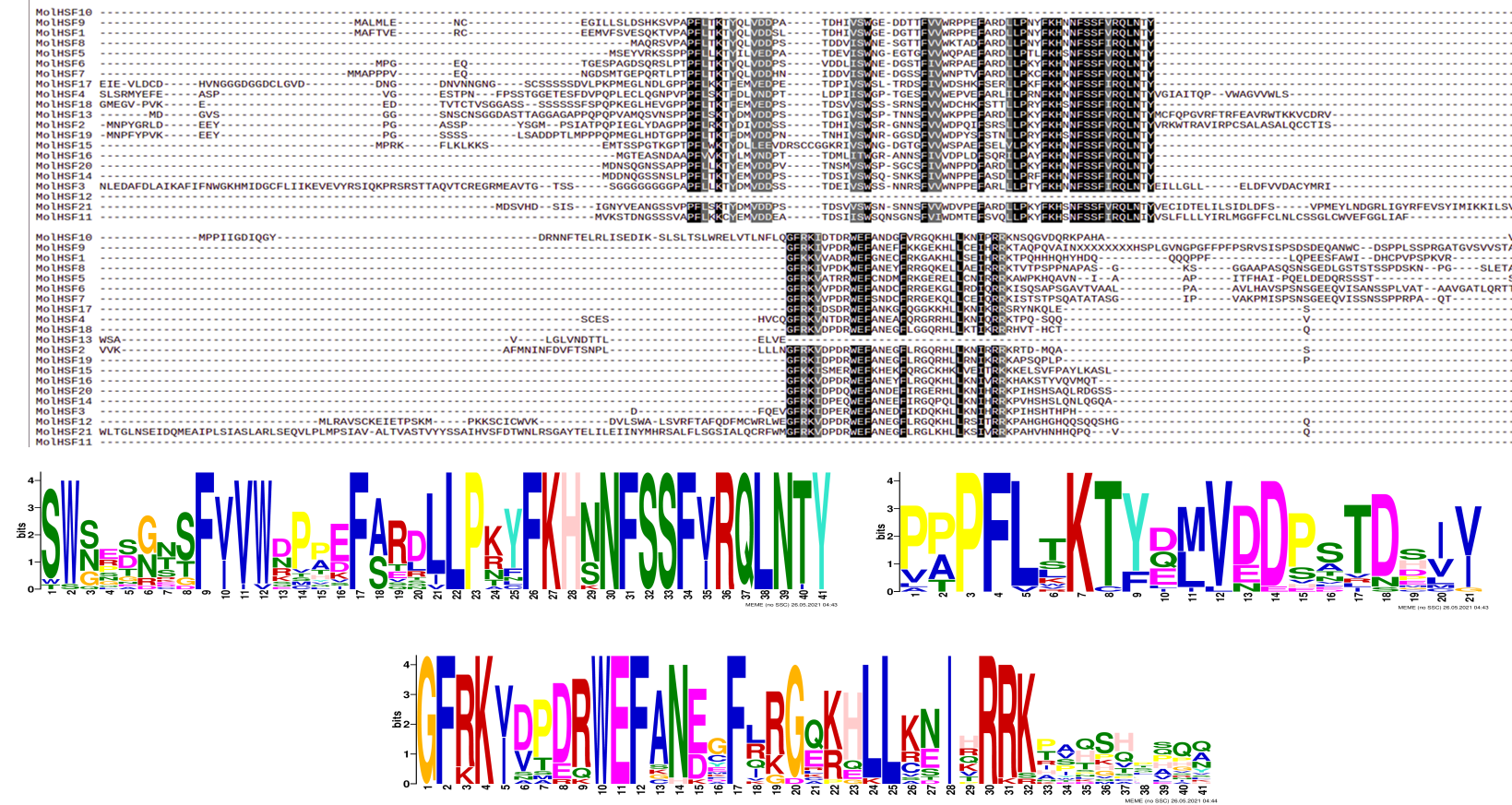

Supplement: Supplementary Figure 1 — Moringa varieties, viz., Bhagya, ODC3, PKM1, and PKM2, during drought stress treatment. [file Data_Sheet_1.zip › Supplementary Figure 3.TIF]

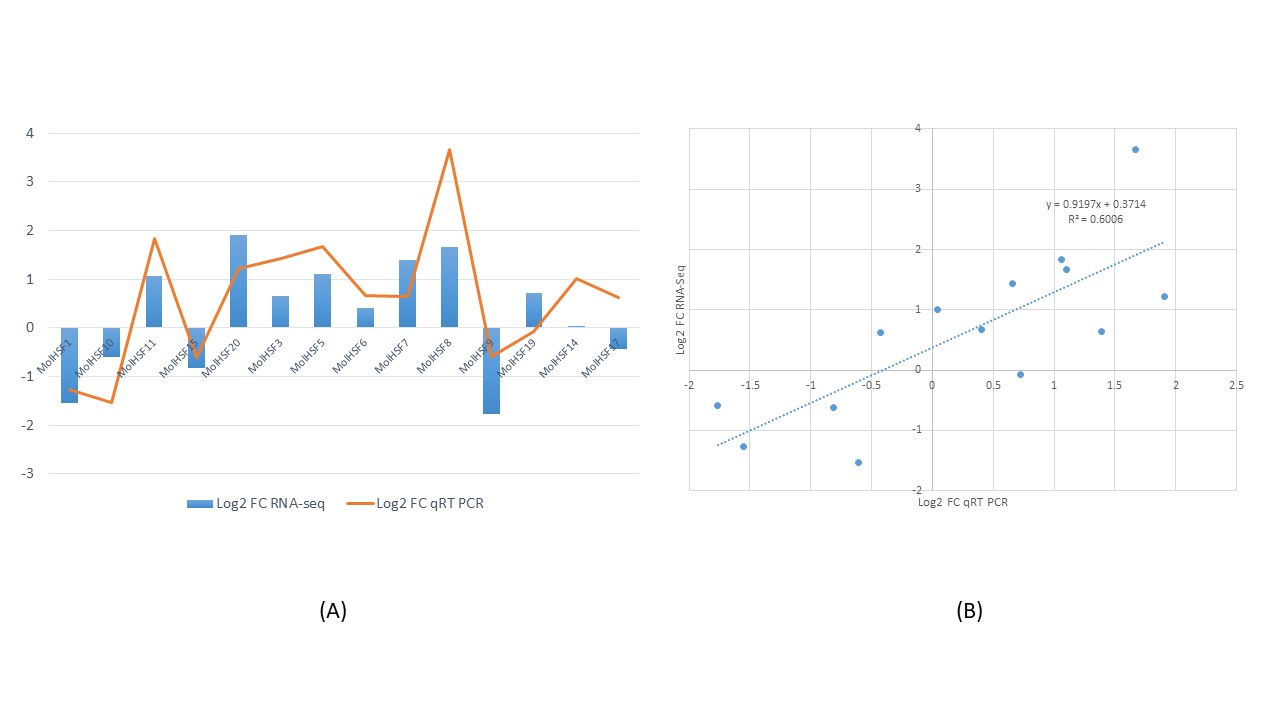

Supplement: Supplementary Figure 1 — Moringa varieties, viz., Bhagya, ODC3, PKM1, and PKM2, during drought stress treatment. [file Data_Sheet_1.zip › Supplementary Figure 4.JPEG]
